# New Sulphated Flavonoids from *Wissadula periplocifolia* (L.) C. Presl (Malvaceae)

**DOI:** 10.3390/molecules201119685

**Published:** 2015-11-09

**Authors:** Yanna C. F. Teles, Carolina Campolina Rebello Horta, Maria de Fátima Agra, Weam Siheri, Marie Boyd, John O. Igoli, Alexander I. Gray, Maria de Fátima Vanderlei de Souza

**Affiliations:** 1 Post-Graduation Program in Development and Technological Innovation in Medicines, Health Sciences Center, Federal University of Paraiba, 58051-900 João Pessoa, PB, Brazil; yannateles@gmail.com; 2 Capes Foundation, Ministry of Education of Brazil, Caixa Postal 250, 70359-970 Brasília, DF, Brazil; carolinacampolina@yahoo.com.br; 3 Biotechnology Center, Federal University of Paraiba, 58051-900 João Pessoa, PB, Brazil; agramf@ltf.ufpb.br; 4 Strathclyde Institute of Pharmacy and Biomedical Sciences, University of Strathclyde, 161 Cathedral Street, G4 0RE Glasgow, UK; weam.siheri@strath.ac.uk (W.S.); marie.boyd@strath.ac.uk (M.B.); john.igoli@strath.ac.uk (J.O.I.); a.i.gray@strath.ac.uk (A.I.G.)

**Keywords:** *Wissadula periplocifolia*, sulphated flavonoids, isoscutellarein derivatives

## Abstract

*Wissadula periplocifolia* (L.) C. Presl (Malvaceae) is commonly used in Brazil to treat bee stings and as an antiseptic. The antioxidant properties of its extracts have been previously demonstrated, thus justifying a phytochemical investigation for its bioactive phenolic constituents. This has yielded five new sulphated flavonoids: 8-*O*-sulphate isoscutellarein (yannin) (**1a**); 4′-*O*-methyl-7-*O*-sulphate isoscutellarein (beltraonin) (**1b**); 7-*O*-sulphate acacetin (wissadulin) (**2a**); 4′-*O*-methyl-8-*O*-sulphate isoscutellarein (caicoine) (**2b**) and 3′-*O*-methyl-8-*O*-sulphate hypolaetin (pedroin) (**3b**) along with the known flavonoids 7,4′-di-*O*-methyl-8-*O*-sulphate isoscutellarein (**4**), acacetin, apigenin, isoscutellarein, 4′-*O*-methyl isoscutellarein, 7,4′-di-*O*-methylisoscutellarein, astragalin and tiliroside. The compounds were isolated by column chromatography and identified by NMR (^1^H, ^13^C, HMQC, HMBC and COSY) and LC-HRMS. A cell based assay was carried out to evaluate the preliminary cytotoxic properties of the flavonoids against UVW glioma and PC-3M prostate cancer cells as well as non-tumour cell lines. The obtained results showed that acacetin, tiliroside, a mixture of acacetin + apigenin and the sulphated flavonoids **2a** + **2b** exhibited inhibitory activity against at least one of the cell lines tested. Among the tested flavonoids acacetin and tiliroside showed lower IC_50_ values, presenting promising antitumor effects.

## 1. Introduction

The Malvaceae family is estimated to contain 243 genera with 4225 species. They have a cosmopolitan distribution and are predominant in the tropics [1]. The family is known to be rich in flavonoids [2,3,4] and sulphur compounds have been reported from a few species such as *Malva sylvestris* L. and *Sidastrum micranthum* (A. St.-Hil.) Fryxell [5,6,7].

Sulphated flavonoids represent an uncommon group of interesting compounds found in some plant families such as Asteraceae, Bixaceae, Dilleniaceae and Verbenaceae [8,9]. These compounds are usually single sulphate or multi-sulphate esters of known flavonoids. The first reported sulphated flavonoid, isorhamnetin 3-sulphate, was isolated in 1937 from *Polygonum hydropiper* L. (Polygonaceae) found in swampy areas of Europe [10]. Thus it was demonstrated that a strong correlation exists between plants growing in aquatic habitats rich in mineral salts and the synthesis of sulphated flavonoids. Therefore, the sulphation of flavonoids could be considered as a result of an ecological adaptation [11,12]. Sulphated flavonoids seem to have an important role in regulation of plant growth and co-pigmentation by forming stable complexes with anthocyanin pigments [13,14]. Sulphation is also considered a detoxification pathway, but in the plant kingdom many other biological functions related to this transformation continue to be discovered including molecular recognition and signaling pathways [15]. The transfer of the functional sulphur group to hydroxylated substrates is catalyzed by a family of sulphotransferase isoforms (SOT). The SOT-catalyzed sulphation requires 3′-phosphoadenosine 5′-phosphosulphate as the sulphate donor and compounds with free hydroxyl groups, *i.e.*, flavonoids, as acceptors [16]. Several sulphated flavonoids have already been described for their antiviral and anticoagulant activities [17,18,19].

*Wissadula periplocifolia* (L.) C. Presl (Malvaceae) is known in Brazil as “malva amarela” and is used to treat bee stings and as an antiseptic [20]. Previous studies have demonstrated its great antioxidant potential, thus justifying a phytochemical examination for phenolic compounds [4]. This study reports the phytochemical investigation of *W. periplocifolia* and the isolation of new sulphated flavonoids along with known flavonoids. In addition, cytotoxic properties of the compounds were evaluated.

## 2. Results and Discussion

### 2.1. Structure Elucidation of Compounds

Chromatographic procedures led to the isolation of flavonoids from aerial parts of *W. periplocifolia*. The compounds were identified by analyzing their 1D and 2D NMR data, and confirmed by their accurate masses and molecular formulas obtained with LC-HRMS.

**Figure 1:**
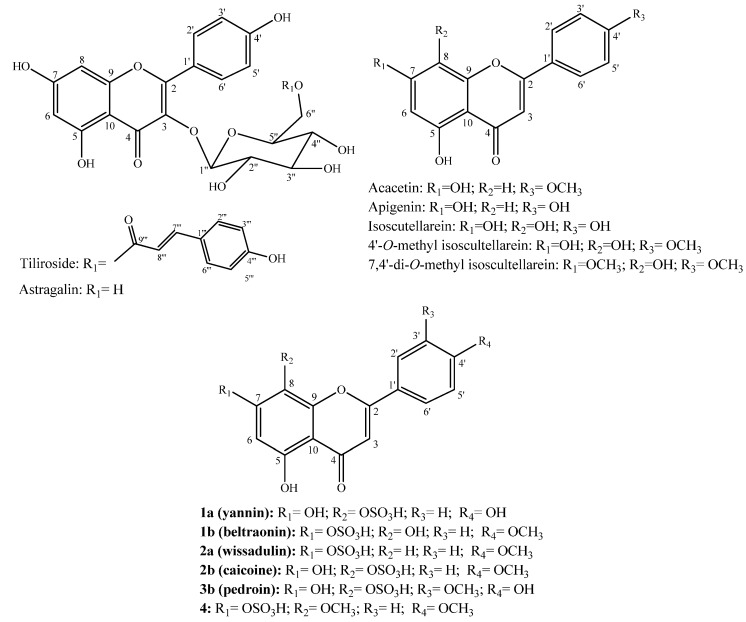
Compounds isolated from *Wissadula periplocifolia*.

The ^1^H-NMR of **1** showed a complex set of signals in the δ_H_ 6 to 8 ppm range and the presence of two downfield singlets at δ_H_ 12.75 and δ_H_ 12.16, characteristic of flavonoids with H-bonded hydroxyl proton at C-5 and the possibility of being a mixture of two flavonoids. The ^13^C-NMR spectrum showed 31 signals and using the HMBC, HMQC and COSY spectra it was possible to identify the compounds in the mixture. The major constituent **1a** showed singlets at δ_H_ 6.81 attached to C-3 and at δ_H_ 6.29 attached to C-6 (HSQC). Two doublets at δ_H_ 8.03 (2H, *J* = 8.74 Hz) and δ_H_ 6.93 (2H, *J* = 8.75 Hz) indicated a *para*-substituted B ring with a scaffold similar to isoscutellarein [21] (Table 1). However, comparison with isoscutellarein ^13^C-NMR data showed that for compound **1a** the signal for C-8 is shielded by 4 ppm while C-7, C-9 and C-5 were deshielded by about 4 ppm. Besides, **1a** was found to be more polar than isoscutellarein, moving slower on TLC. These facts suggest an *O*-sulphate group attached at C-8 instead of a hydroxyl as for isoscutellarein. These chemical shifts are usually observed for *O*-sulphate flavonoids [17,22]. The presence of *O*-sulphate group was confirmed by LC-HRMS. The minor constituent **1b** showed singlets at δ_H_ 6.93 attached to C-3 and δ_H_ 6.90 attached to C-6, two doublets at δ_H_ 8.11 (2H, *J* = 8.88 Hz) and δ_H_ 7.14 (2H, *J* = 8.90 Hz) and a methoxyl at δ_H_ 3.86 (Table 1). The HMBC showed a strong correlation of the methoxyl with a carbon at δ_c_ 163.0, confirming the methoxyl to be at C-4′. Comparison of the NMR data of **1b** and 4′-*O*-methylisoscutellarein [23] indicated that position 7 was shielded by 5 ppm, and positions C-6 and C-8 were found to be deshielded by 4 and 6 ppm. Like compound **1a,** an *O*-sulphate substitution is proposed, but for **1b** this group is found at C-7. In order to confirm the *O*-sulphate group in **1a** and **1b**, the HRMS of the compounds were obtained by LC-HRMS. The accurate mass (molecular formula) for compound **1a** as [M − H]^−^ ion, found at retention time (RT) of 5.34 min, was 365.0049 (C_15_H_9_O_9_S) and for compound **1b** also as an [M − H]^−^ ion (RT: 6.05 min) was 379.0206 (C_16_H_11_O_9_S). These results confirm the presence of *O*-sulphate groups in both molecules. Thus, compound **1a** was identified as 8-*O*-sulphate isoscutellarein (yannin) and compound **1b** as 4′-*O*-methyl-7-*O*-sulphate isoscutellarein (beltraonin) (Figure 1) and are hereby reported for the first time.

**Table 1:** NMR data (DMSO-*d*_6_, ^1^H 400 MHz and ^13^C 100 MHz) of *O*-sulphated flavonoids from *Wissadula periplocifolia*.

Position	1a		1b		2a		2b		3b		4	
	δ_H_ (*J* in Hz)	δ_C_	δ_H_ (*J* in Hz)	δ_C_	δ_H_ (*J* in Hz)	δ_C_	δ_H_ (*J* in Hz)	δ_C_	δ_H_ (*J* in Hz)	δ_C_	δ_H_ (*J* in Hz)	δ_C_
2	-	164.5, C	-	164.3, C	-	164.3, C	-	163.9, C	-	164.4, C	-	164.4, C
3	6.81, s	103.1, CH	6.93, s	103.9, CH	6.93, s	104.2, CH	6.89, s	103.7, CH	6.87, s	103.3, CH	6.87, s	103.3, CH
4	-	182.5, C	-	183.1, C	-	182.6, C	-	182.4, C	-	182.5, C	-	182.8, C
5	-	157.5, C	-	152.0, C	-	161.0, C	-	157.4, C	-	157.5, C	-	157.6, C
6	6.29, s	100.0, CH	6.90, s	104.1, CH	6.60, d (1.8)	102.1, CH	6.30, s	100.0, CH	6.29, s	99.9, CH	6.53, s	96.6, CH
7	-	157.5, C	-	148.3, C	-	160.1, C	-	157.5, C	-	157.5, C	-	159.7, C
8	-	121.9, C	-	129.5, C	7.05, d (1.8)	98.0, CH	-	121.9, C	-	121.1, C	-	123.3, C
9	-	150.2, C	-	145.7, C	-	156.9, C	-	150.1, C	-	151.9, C	-	149.9, C
10	-	104.4, C	-	106.8, C	-	106.2, C	-	104.4, C	-	104.4, C	-	104.3, C
1′	-	121.9, C	-	123.5, C	-	123.1, C	-	123.4, C	-	122.3, C	-	123.7, C
2′	8.03, d (8.7)	129.0, CH	8.11, d (8.9)	129.3, CH	8.07, d (8.7)	129.0, CH	8.14, d (8.9)	129.1, CH	7.64, dd (1.8, 8.5)	121.1, CH	8.28, d (8.7)	129.7, CH
3′	6.93, d (8.7)	116.4, CH	7.14, d (8.9)	115.2, CH	7.12, d (8.7)	115.1, CH	7.13, d (8.9)	115.0, CH	7.14, dd (0.7, 8.5)	115.2, CH	7.08, d (8.7)	114.9, CH
4′	-	161.8, C	-	163.0, C	-	162.9, C	-	162.9, C	-	151.2, C	-	162.8, C
5′	6.93, d (8.7)	116.4, CH	7.14, d (8.9)	115.2, CH	7.12, d (8.7)	115.1, CH	7.13, d (8.9)	115.0, CH	-	148.5, C	7.08, d (8.7)	114.9, CH
6′	8.03, d (8.7)	129.0, CH	8.11, d (8.9)	129.3, CH	8.07, d (8.7)	129.0, CH	8.14, d (8.9)	129.1, CH	7.82, d (1.8)	111.2, CH	8.28, d (8.7)	129.7, CH
OCH_3_-4′	-	-	3.86, s	56.1, CH_3_	3.86, s	56.0, CH_3_	3.87, s	56.0, CH_3_	-	-	3.86, s	56.1, CH_3_
OCH_3_-7	-	-	-	-	-	-	-	-	-	-	3.85, s	57.0, CH_3_
OCH_3_-5′	-	-	-	-	-	-	-	-	3.88, s	56.4, CH_3_	-	-
OH-5	12.75, s	-	12.16, s	-	12.82, s	-	12.70, s	-	12.71, s	-	12.87, s	-
OH-8	-	-	8.98, s	-	-	-	-	-	-	-	-	-
OH-7	9.96, s	-	-	-	-	-	-	-	-	-	-	-
OH-4′	10.42, s	-	-	-	-	-	-	-	-	-	-	-

The ^1^H-NMR of **2** showed a similar pattern to **1**. There were also two downfield singlets at δ_H_ 12.82 and δ_H_ 12.70 integrating for 1.0 and 0.51, respectively. The set of aromatic protons also indicated that **2** was a mixture of two flavonoids. Additionally, a signal corresponding to methoxyl protons was integrated for 4.61, suggesting the presence of one methoxyl for each flavonoid in the mixture. The ^13^C-NMR spectra also showed 31 signals including a duplicated methoxyl carbon at δ_c_ 56.03. By analyzing the HMBC and HMQC spectra of this mixture, it was possible to determine the major constituent (compound **2a**) to possess an acacetin-like moiety and the minor one (compound **2b**) showed a 4′-*O*-methyl isoscutellarein skeleton. However, comparison of **2a** with acacetin NMR data indicated that C-7 in compound **2a** is shielded by 4 ppm and C-6, C-8 and C-10 are deshielded by 3 ppm [3]. This is the same chemical shift difference observed for compound **1b**, indicating that compound **2a** is substituted by an *O*-sulphate at C-7. Comparison of the NMR data of compound **2b** with 4′-*O*-methyl isoscutellarein also indicated some chemical shift differences: C-8 is shielded by 4 ppm while C-7, C-5 and C-9 were deshielded by 4 ppm, thus compound **2b** may possess an *O*-sulphate at C-8 just like **1a** [23]. The accurate masses obtained for the [M − H]^−^ ions were 363.0258 (RT: 5.20 min) (**2a**) and 379.0204 (RT: 6.50 min) (**2b**), and the molecular formulas found were C_16_H_11_O_8_S and C_16_H_11_O_9_S respectively. Compound **2a** was identified as 7-*O*-sulphate acacetin (wissadulin) and compound **2b** was identified as 4′-*O*-methyl-8-*O*-sulphate isoscutellarein (caicoine) (Figure 1), both reported for the first time.

Similar to the mixtures of **1** and **2**, the ^1^H-NMR of **3** indicated the presence of a mixture of two flavonoids. The two downfield singlets were found at δ_H_ 12.75 and δ_H_ 12.71 integrated for 1.0 and 0.47 respectively. Using the NMR data of **3** the major compound of this mixture **3a** was found to be similar to compound **1a** (yannin). The minor constituent of the mixture (compound **3b**) showed two singlets at δ_H_ 6.87 and δ_H_ 6.29 integrated for 1H each and another singlet at δ_H_ 3.88 integrated for 3H suggesting the presence of a methoxyl group. A set of doublets at δ_H_ 7.82 (1H; *d*, *J* = 1.8 Hz), δ_H_ 7.64 (1H; *dd*, *J* = 1.8 and 8.5 Hz) and δ_H_ 7.14 (1H; *dd*, *J* = 0.7 and 8.5 Hz), indicated that ring B is substituted at positions 3′ and 4′. From the HMBC and HMQC spectrum of this mixture it was possible to determine that **3b** possesses a 3′-*O*-methylhypoaletin skeleton [24]. When compared to literature NMR data, **3b** showed that C-8 was more shielded while C-7 and C-9 were deshielded, indicating that **3b** may have an *O*-sulphate group at C-8 of a 3′-*O*-methylhypoaletin skeleton. The accurate mass and the molecular formula of compound **3b** [M − H]^−^ ion were obtained as 395.0136 and C_16_H_11_O_10_S (RT 5.41 min), respectively, thus the structure of compound **3b** was determined as 3′-*O*-methyl-8-*O*-sulphate hypoaletin (pedroin) (Figure 1) and it is also being reported for the first time.

Compound **4** showed a skeleton similar to 7,4′-di-*O*-methylisoscutellarein [3]. As observed for the other *O*-sulphated flavonoids, differences in chemical shifts were found for C-8 which was 3 ppm shielded while C-7 and C-9 were 4 ppm deshielded as in **1a**, **2b** and **3b**. The accurate mass obtained for the [M − H]^+^ ion (RT 6.15 min) of compound **4** was 395.0405 and the molecular formula C_17_H_15_O_9_S allowed its identity to be confirmed as 7,4′-di-*O*-methyl-8-*O*-sulphate isoscutellarein (Figure 1), previously reported from *Sidastrum micranthum* (Malvaceae) [7].

The non-sulphated flavonoids isolated from *W. periplocifolia* were identified by comparisons of their NMR data with literature (see Experimental). They are being reported for the first time from *Wissadula* genera.

### 2.2. Evaluation of Cytotoxicity

The cytotoxic evaluation of the flavonoids from *W. periplocifolia* against tumour (UVW, PC-3M) and non-tumour (PNT2A, Hs27) cell lines was accomplished by the alamarBlue assay. Among all tested fractions, only acacetin, tiliroside, a mixture of acacetin + apigenin and the sulphated flavonoids **2a** + **2b** exhibited inhibitory activity against at least one of the cell lines tested. Table 2 shows the 50% inhibitory concentrations (IC_50_) of the tested samples on each cell line. Comparison between IC_50_ values shows that the treatment with these flavonoids (0.4–100 µg/mL) for 24 h exerted higher anti-proliferative activity against tumour cells (mainly PC-3M prostate carcinoma cells), when compared to both non-cancer cell lines.

**Table 2:** IC_50_ for 24h treatment with *Wissadula periplocifolia* compounds in different cell lines.

Treatment	IC_50_ (µg/mL)			
	Tumour Cell Lines		Normal Cell Lines	
	UVW	PC-3M	PNT2A	Hs27
acacetin	27.37 ± 1.09 ^ab^	21.13 ± 1.16 ^ab^	51.33 ± 1.09	>100
7,4′-di-*O*-methylisoscutellarein	98.55 ± 1.13 ^ab^	>100	88.73 ± 1.13	NA
tiliroside	>100	60.55 ± 1.12 ^ab^	>100	NA
acacetin + apigenin (1.2:1)	>100	48.13 ± 1.09 ^ab^	>100	>100
**2a** + **2b** (2:1)	>100	92.14 ± 1.09 ^ab^	>100	NA

The values are means ± SD of at least three independent experiments performed in duplicates. NA = not applicable. ^a^ *p* < 0.05 compared to PNT2A; ^b^ *p* < 0.05 compared to Hs27.

Among the flavonoids from *W. periplocifolia* tested, 7,4′-di-*O*-methylisoscutellarein and the sulphated flavonoids **2a** + **2b** presented anti-proliferative effects against UVW and PC-3M cells, respectively, but with higher IC_50_ values. Acacetin exhibited the best cytotoxicity against UVW and PC-3M cells, with the lowest IC_50_ values. Most importantly, the anti-proliferative effects of acacetin were higher in tumour cells when compared to non-cancer cell lines. It has been shown that acacetin exhibits anti-proliferative activities against many tumour cell lines [25,26,27], including glioma and prostate carcinoma cells. However, its underlying mechanisms of action remain unknown. In glioma cells, acacetin inhibits the production of TGF-β1, an angiogenic cytokine [28]. In prostate cancer cells, acacetin effects occur via apoptotic pathways that target Akt/NF-κB signalling [29], inhibit JAK1/2 and STAT3 signaling [30], or is accompanied by poly-(ADP-ribose) polymerase cleavage. It can act through inhibition of cell cycle progression as well. In addition, in DU145 human prostate carcinoma cell line, the treatment with acacetin induces down regulation of the expression of matrix metalloproteinase-2 (MMP-2), matrix metalloproteinase-9 (MMP-9), and urokinase-type plasminogen activator (u-PA) through suppressing p38 MAPK signaling pathway, proving that it might also be used as an antimetastatic agent [31].

Apigenin is another flavone that decreases viability of many cancer cells through mechanisms that had not been completely explored so far [32,33,34,35,36,37]. Nevertheless, in our assays, apigenin did not present a synergistic effect with acacetin. The mixture of acacetin + apigenin (1.2:1) was not toxic to UVW cells and exhibited lower inhibitory activity against PC-3M cells compared to treatment with apigenin alone.

Tiliroside isolated from *W. periplocifolia* exerted selective anti-proliferative activity against PC-3M prostate carcinoma cells, when compared to glioma and both non-cancer cell lines. Tiliroside is a naturally occurring flavonoid that has toxic effects on some tumour cell lines such as lung cancer A549 [38] and no significant cytotoxic action on many other cell lines such as DMS114, H460, MCF7, MB435, DU145, SF268, HT29, HCT116, NCI-H292, HEp-2 and KB cells [39,40]. In human endometrial carcinoma cells that were subjected to oxidative stress, the treatment with tiliroside could restore all the alterations on the cells to the control level, thereby showing an antioxidative action of this flavonoid through insulin-like growth factor-I receptor (IGF-IR) signalling [41]. However, the peracetylated derivate of tiliroside presents anti-proliferative action on many tumour cells. Thus, the strategy of peracetylation improves its cytotoxic effects [40,42]. Tiliroside significantly inhibited the growth of sarcoma 180 and carcinoma of Ehrlich tumours that were implanted in mice, even though the flavonoid was not toxic to some tumour cell lines tested *in vitro*. Therefore, tiliroside presents promising antitumor effects without a significant toxicity [39].

In this study, we showed preliminary cytotoxic characterizations of flavonoids isolated from *W. periplocifolia* on glioma and prostate cancer cell lines. Further analyses are necessary to investigate the mechanisms of action of these compounds.

## 3. Experimental Section

### 3.1. General Procedures

Column chromatography separations (CC) were performed on glass columns packed with silica gel (ASTM, 230-400 mesh, Merck, Nottingham, UK) and gel filtration chromatography (GFC) were carried out using Sephadex LH-20 (Sigma-Aldrich, Irvine, UK). Thin layer chromatography (TLC) were performed on silica gel PF 254 plates (Merck, Nottingham, UK) and spots were visualized under UV light (254 and 366 nm) and by spraying with vanillin–sulphuric acid reagent. Isolated compounds were identified by 1D and 2D NMR analysis (^1^H 400 MHz, ^13^C 100 MHz), acquired on a Bruker-Avance III spectrometer (Bruker, Coventry, UK) using deuterated DMSO.

### 3.2. Plant Material

The aerial parts of *W. periplocifolia* were collected in Araruna City, Paraiba/Brazil (GPS coordinates 6°27′29′′S 35°40′43′′W), in August 2005 (SISBIO Authorization Number 46923-2). A voucher specimen (JPB 6498) was authenticated by Prof. Dr. Maria de Fátima Agra and deposited at Prof. Lauro Pires Xavier Herbarium (JPB/UFPB).

### 3.3. Extraction and Isolation

The plant material was dried in an oven at 40 °C for 72 h. After milling, 8.9 kg of powder was macerated with absolute ethanol for 72 h. The obtained ethanol extract was concentrated with a rotatory evaporator yielding 705 g of crude extract (CEE). 200 g of CEE was solvent extracted using hexane, dichloromethane (CH_2_Cl_2_), ethyl acetate (EtOAc) and *n*-butanol to yield 48 g of hexane (HF), 33 g of CH_2_Cl_2_ (DF), 28 g of EtOAc (EAF) and 9 g of *n*-butanol (BF) fractions. The various fractions were analysed by ^1^H-NMR to detect the presence of phenolic constituents and DP, EAP and BF were chosen for column chromatography (CC).

The fraction DF (15 g) was subjected to silica column chromatography (CC) eluted with hexane, EtOAc and methanol in a gradient manner. The fractions were analysed by TLC and interesting fractions were further subjected to GFC, eluted isocratic wise with methanol to isolate the compounds. CC fraction 58–69 chromatographic separation on GFC led to isolation of acacetin (35 mg) and 7,4′-di-*O*-methylisoscutellarein (24 mg), identified by NMR and LC-MS techniques and by comparisons with literature data. CC fraction 79–92 on GFC yielded the compounds apigenin, isoscutellarein, and 4′-*O*-methyl isoscutellarein. Their NMR data match with literature [21,23].

The CC fraction 206–218 (246 mg) was subjected to GFC yielding 34 fractions. These fractions were analyzed by TLC and combined. Fractions 16–17 (12 mg) and 20–21 (9 mg) showed only one spot at TLC, named as compounds **1** and **2** respectively (Figure 1 and Table 1). The CC combined fraction 219–227 (210 mg) was chromatographed under GFC resulting in 35 fractions. Fraction 31–35 (60 mg) was again subjected to GFC yielding 14 fractions. The resulting fractions were analyzed by TLC and the sample 4–10 (10 mg) showed one spot at TLC and it was coded as compound **3** (Figure 1 and Table 1).

EAF (2 g) was subjected to GFC eluted with methanol. Fractions were analyzed by TLC and selected to purification also through GFC. From this process two glucosyl flavonoids were purified: kaempferol 3-*O*-β-D-glucopyranoside (astragalin) (12 mg) and kaempferol-3-*O*-b-d-(6′′-*E-p*-coumaroyl) glucopyranoside (tiliroside) (95 mg). The purified fractions were analyzed by 1D and 2D NMR techniques and by LC-MS and the data match with literature [3,43].

BF (2 g) was subjected to consecutives GFC eluted with methanol. From this process it was purified compound **4** (32 mg) (Figure 1 and Table 1) and the tiliroside, also isolated from EAF.

### 3.4. Compound Identification

The isolated compounds were identified by 1D and 2D NMR analysis (^1^H 400 MHz, ^13^C 100 MHz) using deuterated DMSO, and their mass spectra were obtained by LC-HRMS. The NMR data of compounds **1** to **4** are showed at Table 1. The NMR spectra and HRMS of compounds **1** to **4** can be found at Supplementary Material (Figure S1–S26).

*Acacetin*. ^1^H-NMR (DMSO) δ (ppm): 12.93 (s, 5-OH), 6.88 (s, H-3), 6.20 (d, *J* = 2 Hz, H-6), 6.51 (d, *J* = 2 Hz, H-8), 8.12 (d, *J* = 8.5 Hz, H-2′, H-6′), 7.11 (d, *J* = 8.5 Hz, H-3′, H-5′), 3.86 (s, -OCH_3_-C4′). ^13^C-NMR (DMSO) δ (ppm): 163.8 (C-2), 104.1 (C-3), 182.3 (C-4), 162.0 (C-5), 99.4 (C-6), 164.7 (C-7), 94.60 (C-8), 157.9 (C-9), 104.3 (C-10), 123.3 (C-1′), 128.8 (C-2′, C-6′), 115.1 (C-3′, C-5′), 162.8 (C-4′), 56.1 (-OCH_3_-4′). The ^1^H- and ^13^C-NMR spectral data are consistent with published data [3].

*Apigenin*. ^1^H-NMR (DMSO) δ (ppm): 12.96 (s, 5-OH), 6.78 (s, H-3), 6.19 (d, *J* = 1.8 Hz, H-6), 6.48 (d, *J* = 1.8 Hz, H-8), 7.92 (d, *J* = 8.7 Hz, H-2′, H-6′), 6.93 (d, *J* = 8.7 Hz, H-3′, H-5′). ^13^C-NMR (DMSO) δ (ppm): 164.3 (C-2), 103.4 (C-3), 182.3 (C-4), 162.0 (C-5), 99.4 (C-6), 164.7 (C-7), 94.5 (C-8), 157.9 (C-9), 104.2 (C-10), 121.7 (C-1′), 129.0 (C-2′, C-6′), 116.5 (C-3′, C-5′), 161.7 (C-4′). The ^1^H- and ^13^C-NMR spectral data are consistent with published data [21].

*Isoscutellarein*. ^1^H-NMR (DMSO) δ (ppm): 12.39 (s, 5-OH), 6.74 (s, H-3), 6.27 (s, H-6), 8.01 (dd, *J* = 8.7 and 2.3 Hz, H-2′, H-6′), 6.93 (dd, *J* = 8.7 and 2.3 Hz, H-3′, H-5′). ^13^C-NMR (DMSO) δ (ppm): 164.1 (C-2), 102.9 (C-3), 182.7 (C-4), 153.6 (C-5), 99.2 (C-6), 153.9 (C-7), 125.6 (C-8), 146.0 (C-9), 103.8 (C-10), 121.9 (C-1′), 129.2 (C-2′, C-6′), 116.4 (C-3′, C-5′), 161.7 (C-4′). The ^1^H- and ^13^C-NMR spectral data are consistent with published data [21].

*4′-O-Methyl isoscutellarein*. ^1^H-NMR (DMSO) δ (ppm): 12.40 (s, 5-OH), 6.83 (s, H-3), 6.28 (s, H-6), 8.12 (d, *J* = 8.9 Hz, H-2′, H-6′), 7.12 (dd, *J* = 8.9 Hz, H-3′, H-5′), 3.86 (s, -OCH_3_-C4′). ^13^C-NMR (DMSO) δ (ppm): 163.7 (C-2), 103.6 (C-3), 182.7 (C-4), 153.6 (C-5), 99.2 (C-6), 153.6 (C-7), 125.6 (C-8), 146.0 (C-9), 103.9 (C-10), 123.6 (C-1′), 129.0 (C-2′, C-6′), 115.1 (C-3′, C-5′), 162.8 (C-4′), 56.1(-OCH_3_-4′). The ^1^H- and ^13^C-NMR spectral data are consistent with published data [23].

*7,4′-di-O-Methyl isoscutellarein*. ^1^H-NMR (DMSO) and ^13^C-NMR (DMSO) δ (ppm). Previously reported [44].

*Tiliroside*. ^1^H-NMR (DMSO) δ (ppm): 12.55 (s, 5-OH), 6.13 (*d*, *J* = 2.0 Hz, H-6), 6.38 (*d*, *J* = 2.0 Hz, H-8), 7.97 (*d*, *J* = 8.8 Hz, H-2′/6′), 6.85 (*d*, *J* = 8.8 Hz, H-3′/5′), 5.43 (*d*, *J* = 7.3 Hz, H-1′′), 3.18–3.40 (*m*, H-2′′, 3′′, 4′′, 5′′), 4.02–4.27 (*m*, H-6′′), 7.34 (*d*, *J* = 8.5 Hz, H-2′′′/6′′′), 6.78 (*d*, *J* = 8.5 Hz, H-3′′′/5′′′), 7.31 (*d*, *J* = 16 Hz, H-β), 6.08 (*d*, *J* = 16 Hz, H-α). ^13^C-NMR (DMSO) δ (ppm): 157.1 (C-2), 133.5 (C-3), 177.9 (C-4), 161.7 (C-5), 99.3 (C-6), 164.7 (C-7), 94.2 (C-8), 156.9 (C-9), 102.5 (C-10), 121.3 (C-1′), 130.7 (C-2′/6′), 116.3 (C-3′′′/5′′′), 160.5 (C-4′), 101.4 (C-1′′), 74.8 (C-2′′), 76.7 (C-3′′), 70.5 (C-4′′), 74.6 (C- 5′′), 63.4 (C-6′′), 125.4 (C-1′′′), 131.3 (2′′′ and 6′′′), 115.6 (C-3′′′ and 5′′′), 160.3 (C-4′′′), 145.2 (C-7′′′), 114.1 (C-8′′′), 166.7 (C-9′′′). The ^1^H- and ^13^C-NMR spectral data are consistent with published data [3].

*Astragalin*. ^1^H-NMR (DMSO) δ (ppm): 12.549 (s, 5-OH), 6.20 (*d*, *J* = 2.0 Hz, H-6), 6.00 (*d*, *J* = 2.0 Hz, H-8), 8.01 (*d*, *J* = 8.8 Hz, H-2′/6′), 6.85 (*d*, *J* = 8.8 Hz, H-3′/5′), 5.37 (*d*, *J* = 7.3 Hz, H-1′′), 3.15–3.55 (*m*, H-2′′, 3′′, 4′′, 5′′, 6′′). ^13^C-NMR (DMSO) δ (ppm): 155.6 (C-2), 133.5 (C-3), 177.1 (C-4), 160.5 (C-5), 101.9 (C-6), 163.1 (C-7), 94.9 (C-8), 155.7 (C-9), 103.5 (C-10), 122.7 (C-1′), 131.2 (C-2′/6′), 115.5 (C-3′′′/5′′′), 160.5 (C-4′), 101.9 (C-1′′), 74.7 (C-2′′), 77.9 (C-3′′), 70.3 (C-4′′), 76.9 (C- 5′′), 61.3 (C-6′′). The ^1^H- and ^13^C-NMR spectral data are consistent with published data [43].

### 3.5. Liquid Chromatography-Mass Spectrometry (LC-MS)

In order to confirm the compounds structures, the high-resolution mass spectra was obtained by LC-MS analysis performed on an Accela 600 HPLC system combined with an Exactive (Orbitrap) mass spectrometer from Thermo Fisher Scientific (Bremen, Germany) in negative or positive mode using method developed to separate phenolic compounds [44].

Each sample was dissolved in methanol (HPLC grade) to obtain a final concentration of 1 mg/mL. The injection volume was 20 μL and an ACE C-18 column (150 × 3 mm, 3 µm) from HiChrom (Reading, UK) was used. A flow rate of 300 μL/min and a mobile phase composed of 0.1% formic acid in H_2_O (solvent A) and acetonitrile (solvent B) was used in a gradient mode as summarized in the Table 3. Data were analysed using Xcalibur 2.2 from Thermo Fisher Scientific.

**Table 3:** Gradient method used at LC-HRMS experiment.

Time (min)	A%	B%
0	75	25
15	25	75

### 3.6. Cell lines and Cell Culture

All media and supplements were obtained from Invitrogen (Paisley, UK). The following human cell lines were used in the current study: the human glioma cancer cell line (UVW) previously described [45], the prostate carcinoma cell line (PC-3M), the normal prostate epithelial cell line (PNT2A), and the normal foreskin fibroblast cell line (Hs27) [46]. All cell lines were obtained from in house stocks (UVW) or from stocks purchased from the ATCC (Rockville, MD, USA) and are routinely genetically verified and confirmed as free from mycoplasma contamination. UVW, PNT2A, and Hs27 cells were maintained in Eagle’s Minimum Essential Medium (MEM), RPMI 1640 Medium, and Dulbecco’s Modified Eagle’s Medium (DMEM), respectively. All media were supplemented with 10% *v*/*v* fetal bovine serum, penicillin (100 units/mL), streptomycin (100 μg/mL), Fungizone (2.5 μg/mL of amphotericin B), and L-glutamine (2 mM). The PC-3M cells were cultured in the same MEM medium as described for UVW cells with the addition of 1% *v*/*v* MEM Non-essential Amino Acids Solution, 1% *v*/*v* MEM Vitamin Solution, and 1mM sodium pyruvate. The cells were cultured at 37 °C in a humidified atmosphere of 5% CO_2_.

### 3.7. Evaluation of Cytotoxicity

Each sample tested was dissolved in DMSO at 50 mg/mL and kept at −20 °C until extract preparation was proceeded. For the biological assays, the samples were diluted in culture medium to get the selected concentrations. The toxicity of the flavonoids on UVW, PC-3M, PNT2A, and Hs27 cells was assessed by the fluorometric measurement of metabolic activity using the AlamarBlue assay (Invitrogen) [46]. Briefly, cells were seeded on 96-well culture plates (3.3 × 10^3^cells/well). After 24 h incubation, the medium was replaced by fresh media containing the flavonoid fractions at 0.4, 1.2, 3.7, 11.1, 33.3, and 100 μg/mL or DMSO at 0.5% *v*/*v*, and then cultured for 24 h at 37 °C in a humidified atmosphere of 5% CO_2_. Untreated cells were used as negative controls. After treatment, the culture medium was removed and replaced by fresh media containing alamarBlue (10% *v*/*v*), and the dye was incubated in the plates with the cells for 4 h. Fluorescence was measured on a micro plate fluorescence reader (Spectramax Gemini XS, Molecular Devices, Sunnyvale, CA, USA) using an excitation wavelength of 560 nm and an emission wavelength of 590 nm. Cell viability was calculated and plotted as the percentage of metabolically active cells relative to the control. The 50% inhibitory concentrations (IC_50_) were calculated graphically from the individual concentration–response curves by non-linear curve fitting. Two-way analysis of variance (two-way ANOVA) with Bonferroni post-hoc test was used to compare the 50% inhibitory concentration values. The level of significance was set at *p* < 0.05 and statistical analysis was performed using GraphPad Prism version 5.0 for Windows (GraphPad Software, San Diego, CA, USA).

## 4. Conclusions

The present study has led to the identification of five new sulphated flavonoids from *Wissadula periplocifolia*: 8-*O*-sulphate isoscutellarein (**1a**); 4′′-*O*-methyl-7-*O*-sulphate isoscutellarein (**1b**); 7-*O*-sulphate acacetin (**2a**); 4′-*O*-methyl-8-*O*-sulphate isoscutellarein (**2b**) and 3′-*O*-methyl-8-*O*-sulphate hypolaetin (**3b**) along with the known flavonoids 7,4′-di-*O*-methyl-8-*O*-sulphate isoscutellarein (**4**), acacetin, apigenin, isoscutellarein, 4′-*O*-methyl isoscutellarein, 7,4′-di-*O*-methyl isoscutellarein, astragalin and tiliroside. Besides, the cytotoxic properties of the isolated compounds were evaluated demonstrating the potential cytotoxicity of acacetin, 7,4′-di-*O*-methyl isoscutellarein, apigenin and the new compounds 7-*O*-sulphate acacetin (**2a**) and 4′-*O*-methyl-8-*O*-sulphate isoscutellarein (**2b**).

## Supplementary Materials

The NMR and HRMS spectra of compounds **1** to **4** are available online at http://www.mdpi.com/1420-3049/20/11/19685/s1.

## References

[1] Rocha J.F., Neves L.J. (2000). Anatomia foliar de *Hibiscus tiliaceus* L. e *Hibiscus*
*pernambucensis* Arruda (Malvaceae). Rodriguesia.

[2] Silva D.A., Falcão-Silva V.S., Gomes A.Y.S., Costa D.A., Lemos V.S., Agra M.F., Braz-Filho R., Siqueira-Junior J.P., Souza M.F.V. (2009). Triterpenes and phenolic compounds isolated from the aerial parts of *Herissantia tiubae* and evaluation of 5,4′,-dihydroxy-3,6,7,8,3′-pentamethoxyflavone as modulator of bacterial drug resistance. Pharm. Biol..

[3] Gomes R.A., Maciel J.K.S., Agra M.F., Souza M.F.V., Falcão-Silva V.S., Siqueira-Junior J.P. (2011). Phenolic compounds from *Sidastrum micranthum* (A. St.-Hil.) Fryxell and evaluation of acacetin and 7,4′-Di-*O*-methylisoscutellarein as motulator of bacterial drug resistence. Quim. Nova.

[4] Oliveira A.M.F., Pinheiro L.S., Pereira C.K.S., Matias W.N., Gomes R.A., Chaves O.S., Souza M.F.V., Almeida R.N., Assis T.S. (2012). Total phenolic content and antioxidant activity of some Malvaceae family species. Antioxidants.

[5] Billeter M., Meier B., Sticher O. (1991). 8-Hydroxyflavonoid glucuronides from *Malva sylvestris*. Phytochemistry..

[6] Nawwar M., Buddrus J. (1981). A gossypetin glucuronide sulphate from the leaves of *Malva sylvestris*. Phytochemistry.

[7] Buchholz H., Wirth C., Carola C., Alves Fontes R. (2007). Flavonoid derivative. U.S. Patent.

[8] Guglielmone H.A., Agnese A.M., Montoya S.C.N., Cabrera J.L. (2005). Inhibitory effects of sulphated flavonoids isolated from *Flaveria bidentis* on platelet aggregation. Thromb. Res..

[9] Gurni A.A., König W.A., Kubitzki K. (1981). Flavonoid glycosides and sulphates from the Dilleniaceae. Phytochemistry.

[10] Harborne J. B. (1975). Flavonoid sulphates: A new class of sulphur compounds in higher plants. Phytochemistry.

[11] Tomás-Barberán F., Harborne J.B., Self R. (1987). *Twelve* 6-Oxygenated-Flavone Sulphates from *Lippia nodiflora* and *L. canescens*. Phytochemistry.

[12] Bylka W., Stobiecki M., Frahski R. (2001). Sulphated flavonoid glycosides from leaves of Atriplex hortensis. Acta Physiol. Plant..

[13] Correia-da-Silva M., Sousa E., Pinto M.M. (2014). Emerging sulfated flavonoids and other polyphenols as drugs: Nature as an inspiration. Med. Res. Rev..

[14] Varin L., Marsolais F., Richard M., Rouleau M. (1997). Sulfation and sulfotransferases 6: Biochemistry and molecular biology of plant sulfotransferases. FASEB J..

[15] Varin L., Marsolais F., Brisson N. (1995). Chimeric flavonol sulfotransferases define a domain responsible for substrate and position specificities. J. Biol. Chem..

[16] Kopriva S., Mugford S.G., Baraniecka P., Lee B., Matthewman C.A., Koprivova A. (2012). Control of sulfur partitioning between primary and secondary metabolism in Arabidopsis. Front. Plant Sci..

[17] Correia-da-Silva M., Sousa E., Duarte B., Marques F., Carvalho F., Cunha-Ribeiro L.M., Pinto M.M. (2011). Flavonoids with an oligopolysulfated moiety: a new class of anticoagulant agents. J. Med. Chem..

[18] Gunnarsson G.T., Desai U.R. (2002). Interaction of designed sulfated flavanoids with antithrombin: Lessons on the design of organic activators. J. Med. Chem..

[19] Liu W., Liang N.-C. (2000). Inhibitory effect of disodium quercetin-7,4′-disulfate on aggregation of pig platelets induced by thrombin and its mechanism. Acta Pharmacol. Sin..

[20] Teles Y.C.F., Gomes R.A., Oliveira M.S., Lucena K.L., Nascimento J.S., Agra M.F., Igoli J.O., Gray A.I., Souza M.F.V. (2014). Phytochemical investigation of *Wissadula periplocifolia* (L.) C. Presl and evaluation of its antibacterial activity. Quim. Nova.

[21] Yoon K.D., Jeong D.G., Hwang Y.H., Ryu J.M., Kim J. (2007). Inhibitors of osteoclast differentiation from *Cephalotaxus koreana*. J. Nat. Prod..

[22] Xia H., Qiu F., Zhu S., Zhang T., Qu G., Yao X. (2007). Isolation and identification of ten metabolites of breviscapine in rat urine. Biol. Pharm. Bull..

[23] Meselhy M.R. (2003). Constituents from Moghat, the roots of *Glossostemon bruguieri* (Desf.). Molecules.

[24] Lin Y., Kong L. (2006). Studies on the chemical constituents of *Desmodium styracifolium* (Osbeck) Merr. Asian J. Tradit. Med..

[25] Hsu Y.L., Kuo P.L., Lin C.C. (2004). Acacetin inhibits the proliferation of Hep G2 by blocking cell cycle progression and inducing apoptosis. Biochem. Pharmacol..

[26] Pan M.H., Lai C.S., Hsu P.C., Wang Y.J. (2005). Acacetin induces apoptosis in human gastric carcinoma cells accompanied by activation of caspase cascades and production of reactive oxygen species. J. Agric. Food Chem..

[27] Shim H.Y., Park J.H., Paik H.D., Nah S.Y., Kim D.S., Han Y.S. (2007). Acacetin-induced apoptosis of human breast cancer MCF-7 cells involves caspase cascade, mitochondria-mediated death signaling and SAPK/JNK1/2-c-Jun activation. Mol. Cells.

[28] Freitas S., Costa S., Azevedo C., Carvalho G., Freire S., Barbosa P., Velozo E., Schaer R., Tardy M., Meyer R., Nascimento I. (2011). Flavonoids inhibit angiogenic cytokine production by human glioma cells. Phytother. Res..

[29] Kim H.R., Park C.G., Jung J.Y. (2014). Acacetin (5,7-dihydroxy-4′-methoxyflavone) exhibits *in vitro* and *in vivo* anticancer activity through the suppression of NF-κB/Akt signaling in prostate cancer cells. Int. J. Mol. Med..

[30] Kim C., Kim M.C., Kim S.M., Nam D., Choi S.H., Kim S.H., Ahn K.S., Lee E.H., Jung S.H., Ahn K.S. (2013). *Chrysanthemum indicum* L. extract induces apoptosis through suppression of constitutive STAT3 activation in human prostate cancer DU145 Cells. Phytother. Res..

[31] Shen K.H., Hung S.H., Yin L.T., Huang C.S., Chao C.H., Liu C.L., Shih Y.W. (2010). Acacetin, a flavonoid, inhibits the invasion and migration of human prostate cancer DU145 cells via inactivation of the p38 MAPK signaling pathway. Mol. Cell. Biochem..

[32] Shukla S., Gupta S. (2010). Apigenin: A promising molecule for cancer prevention. Pharm Res..

[33] Knowles L.M., Zigrossi D.A., Tauber R.A., Hightower C., Milner J.A. (2000). Flavonoids suppress androgen-independent human prostate tumor proliferation. Nutr. Cancer.

[34] Morrissey C., O’Neill A., Spengler B., Christoffel V., Fitzpatrick J.M., Watson R.W. (2005). Apigenin drives the production of reactive oxygen species and initiates a mitochondrial mediated cell death pathway in prostate epithelial cells. Prostate.

[35] Shukla S., Gupta S. (2008). Apigenin-induced prostate cancer cell death is initiated by reactive oxygen species and p53 activation. Free Radic. Biol. Med..

[36] Das A., Banik N.L., Ray S.K. (2010). Flavonoids activated caspases for apoptosis in human glioblastoma t98g and u87mg cells but not in human normal astrocytes. Cancer.

[37] Seibert H., Maser E., Schweda K., Seibert S., Gülden M. (2011). Cytoprotective activity against peroxide-induced oxidative damage and cytotoxicity of flavonoids in C6 rat glioma cells. Food Chem. Toxicol..

[38] Liao C.R., Kuo Y.H., Ho Y.L., Wang C.Y., Yang C.S., Lin C.W., Chang Y.S. (2014). Studies on cytotoxic constituents from the leaves of *Elaeagnus oldhamii* Maxim. In non-small cell lung cancer a549 cells. Molecules.

[39] Carvalho P.R.C., Aguiar J.S., Matias W.N., Gomes R.A., Teles Y.C.F., Souza M.F.V., Medeiros P.L., Silva E.C., Gonçalves-Silva T., Nascimento S.C. (2011). *In vitro* and *in vivo* antitumor effects of the flavonol glycosides isolated of *Herissantia crispa* (L.) Brizicky. Lat. Am. J. Pharm..

[40] Tsimplouli C., Demetzos C., Hadzopoulou-Cladaras M., Pantazis P., Dimas K. (2012). In vitro activity of dietary flavonol congeners against human cancer cell lines. Eur. J. Nutr..

[41] Tomczyk M., Tumanov A., Zaniewska A., Surazynski A. (2010). The potential mechanism of tiliroside-dependent inhibition of t-butylhydroperoxide-induced oxidative stress in endometrial carcinoma cells. Planta Med..

[42] Dimas K., Demetzos C., Vaos B., Marselos M., Kokkinopoulos D. (1999). Cytotoxic and antiproliferative effects of heptaacetyl tilirosid in human leulemic cell lines. Leuk. Res..

[43] Teles Y.C.F., Ribeiro-Filho J., Bozza P.T., Agra M.F., Siheri W., Igoli J.O., Gray A.I., Souza M.F.V. (2015). Phenolic constituents from *Wissadula periplocifolia* (L.) C. Presl. and anti-inflammatory activity of 7,4′-di-*O* -methylisoscutellarein. Nat. Prod. Res..

[44] Beninger C.W., Hosfield G.L., Bassett M.J. (1999). Flavonoid Composition of Three Genotypes of Dry Bean (*Phaseolus. vulgaris*) differing in Seedcoat Color. J. Am. Soc. Hort. Sci..

[45] Boyd M., Mairs R.J., Keith W.N., Ross S.C., Welsh P., Akabani G., Owens J., Vaidyanathan G., Carruthers R., Dorrens J. (2004). An efficient targeted radiotherapy/gene therapy strategy utilising human telomerase promoters and radioastatine and harnessing radiation-mediated bystander effects. J. Gene Med..

[46] Ahmed S.A., Gogal R.M., Walsh J.E. (1994). A new rapid and simple non-radioactive assay to monitor and determine the proliferation of lymphocytes: An alternative to [3H]thymidine incorporation assay. J. Immunol. Methods.

