# Supplementary material for: New Sulphated Flavonoids from Wissadula periplocifolia (L.) C. Presl (Malvaceae)
Source: Molecules. 2015 Nov 9;20(11):20161–72. doi: 10.3390/molecules201119685 (PMC6332353; doi:10.3390/molecules201119685)
Supplement: Supplementary file 1 [file molecules-20-19685-s001.pdf]

# Supplementary Material: New Sulphated Flavonoids from *Wissadula periplocifolia* (L.) C. Presl (Malvaceae)

**Yanna C. F. Teles, Carolina Campolina Rebello Horta, Maria de Fátima Agra, Weam Siheri, Marie Boyd, John O. Igoli, Alexander I. Gray, Maria de Fátima Vanderlei de Souza**

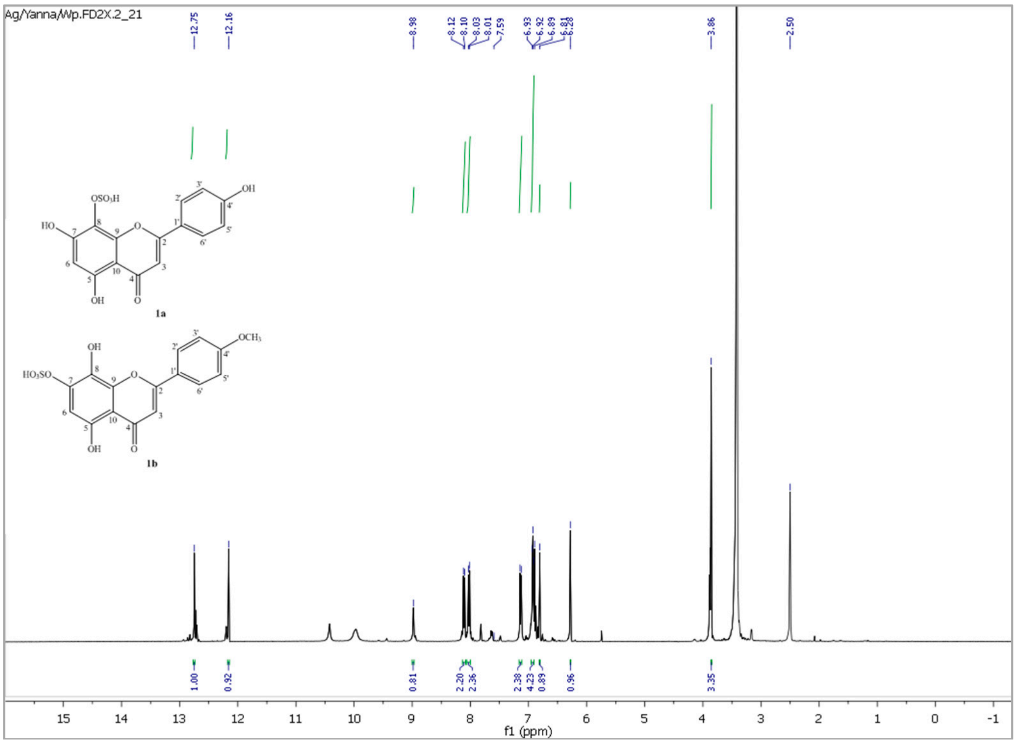


**Figure S1.** $^{1}$H-NMR spectrum (400 MHz, DMSO) of **1a** + **1b**.

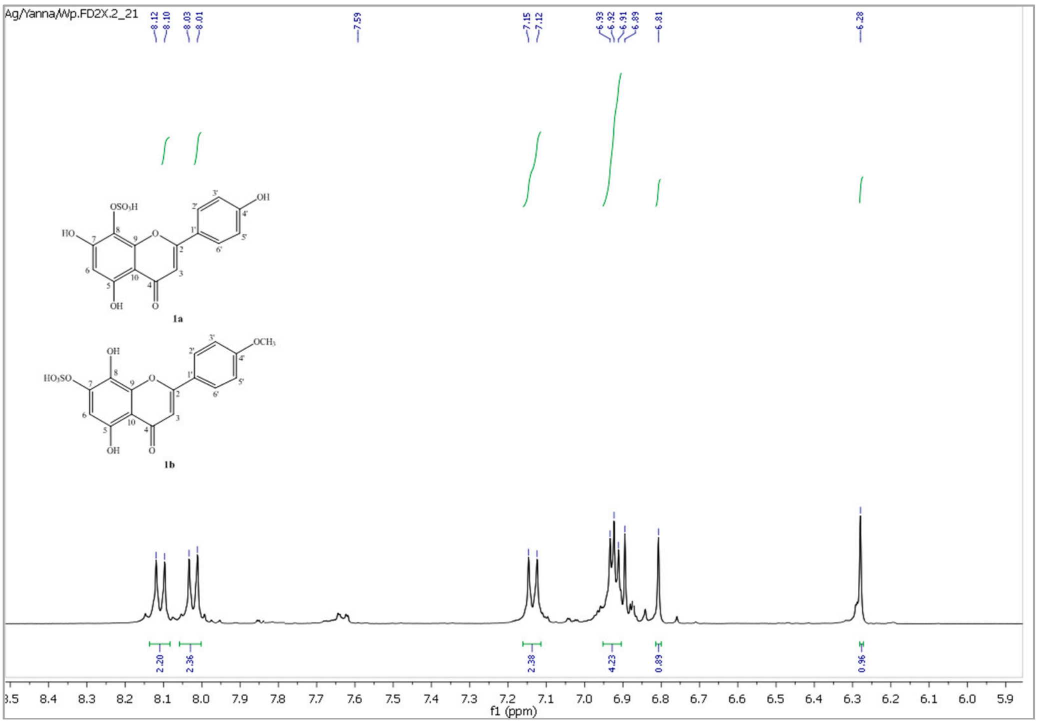


**Figure S2.** Expansion of $^{1}$H-NMR spectrum (400 MHz, DMSO) of **1a** + **1b**.

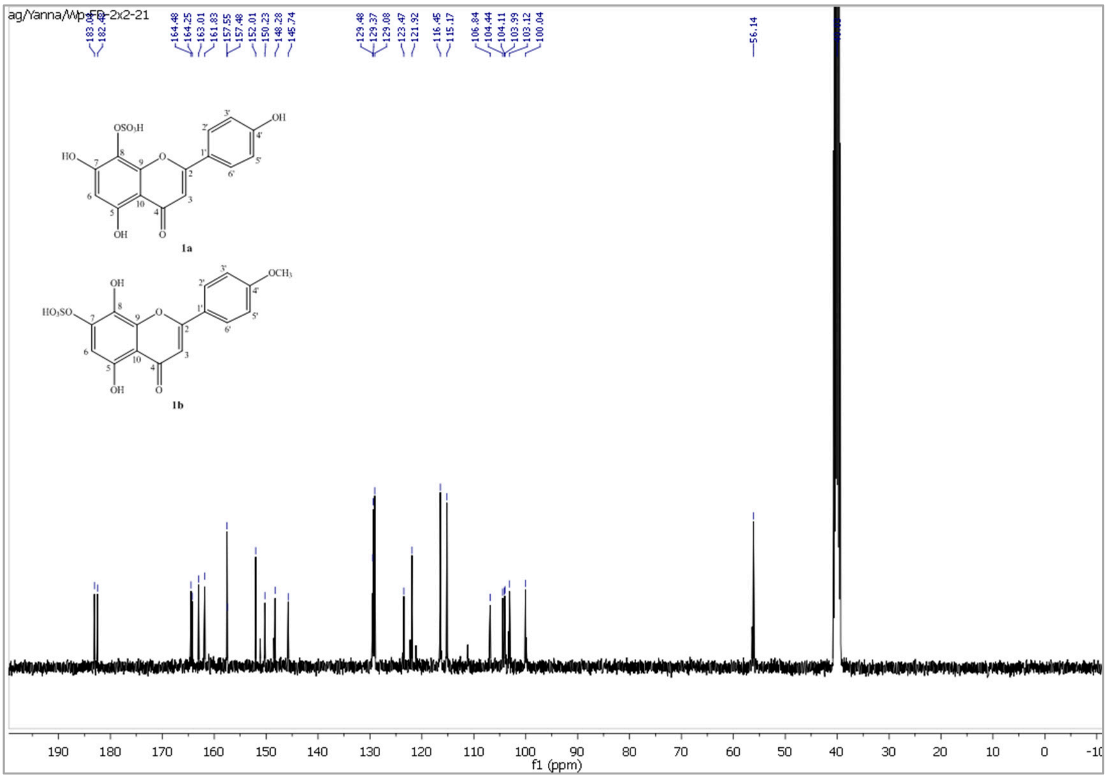


**Figure S3.** $^{13}$C-NMR spectrum (100 MHz, DMSO) of **1a** + **1b**.

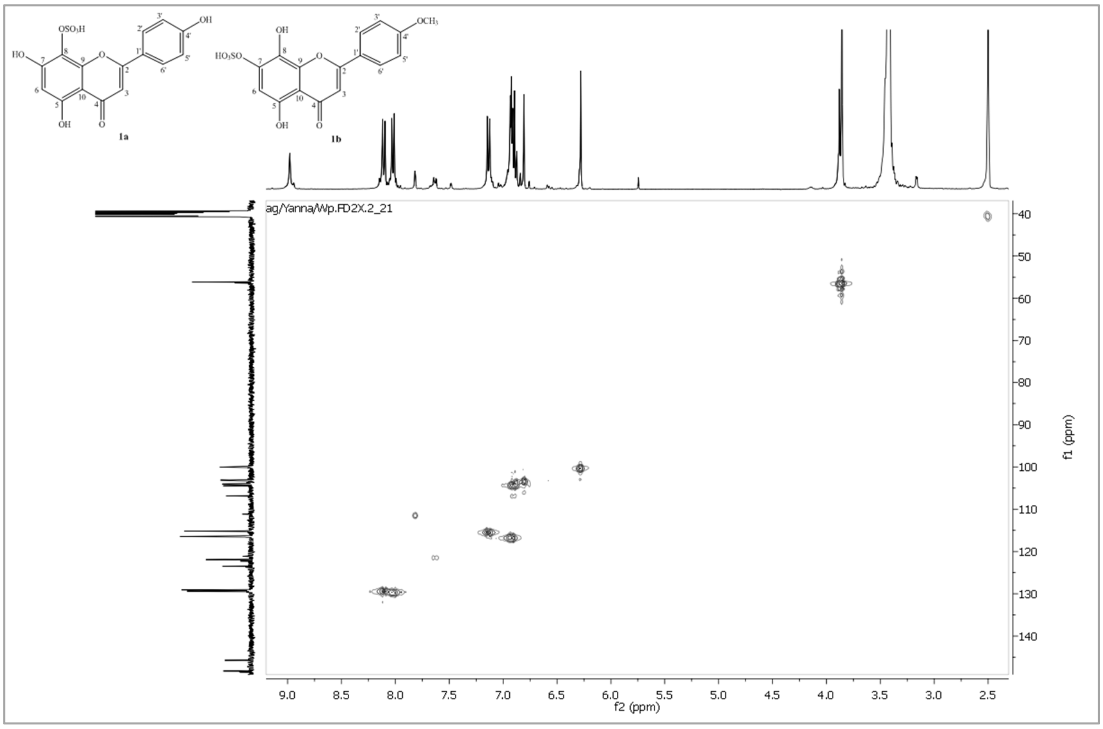


**Figure S4.** HMQC spectrum ($^{1}$H-NMR: 400 MHz, $^{13}$C-NMR: 100 MHz, DMSO) of **1a** + **1b**.

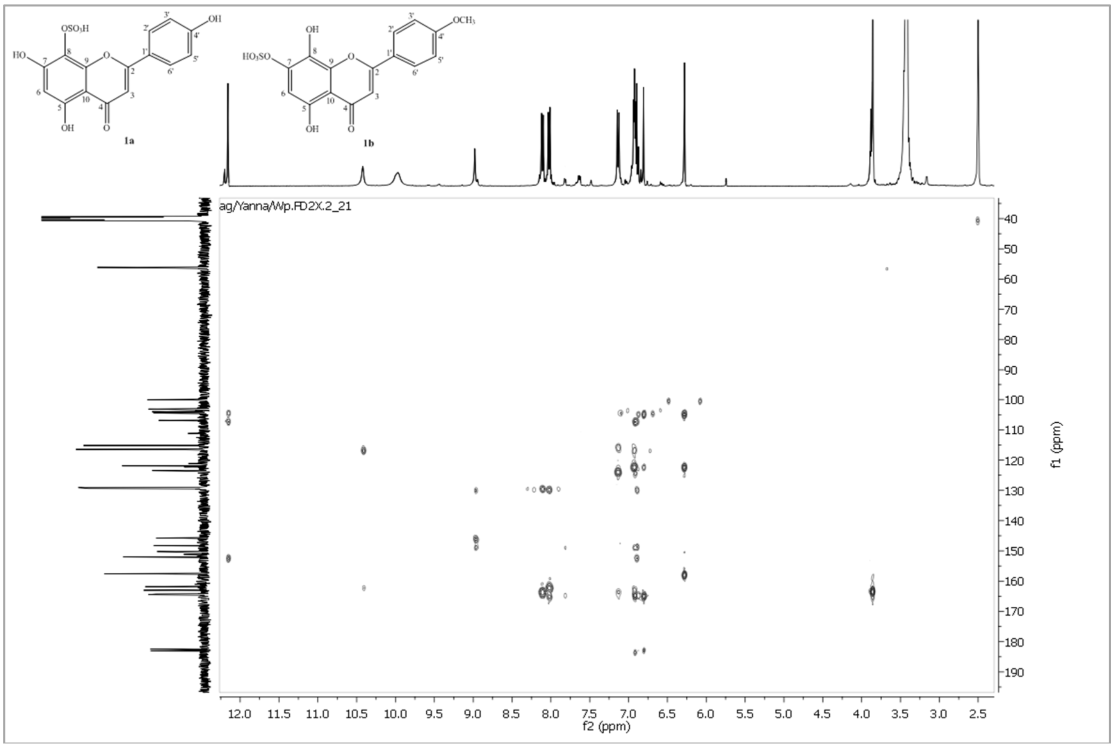


**Figure S5.** HMBC spectrum ($^{1}$H-NMR: 400 MHz, $^{13}$C-NMR: 100 MHz, DMSO) of **1a** + **1b**.

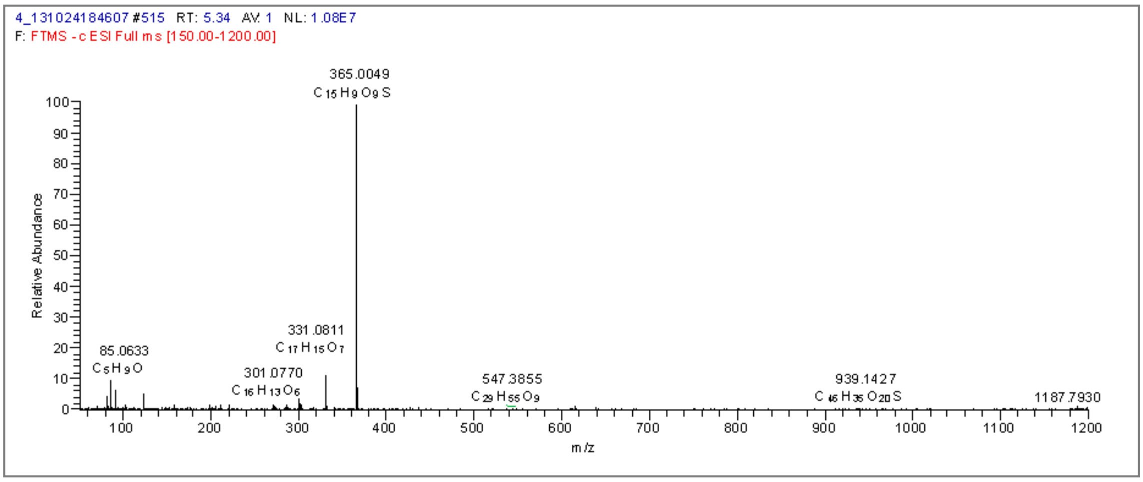


**Figure S6.** HRMS spectrum of compound **1a**.

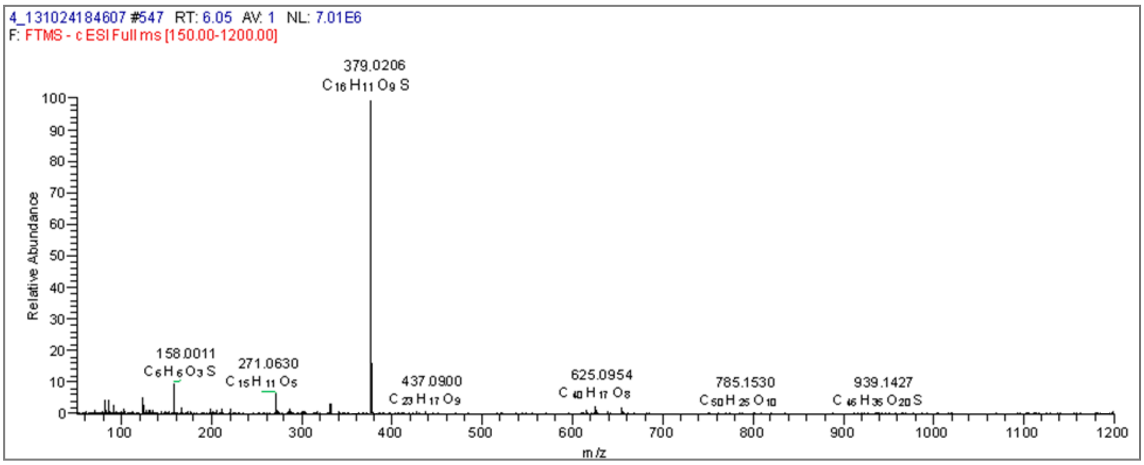


**Figure S7.** HRMS spectrum of compound **1b**.

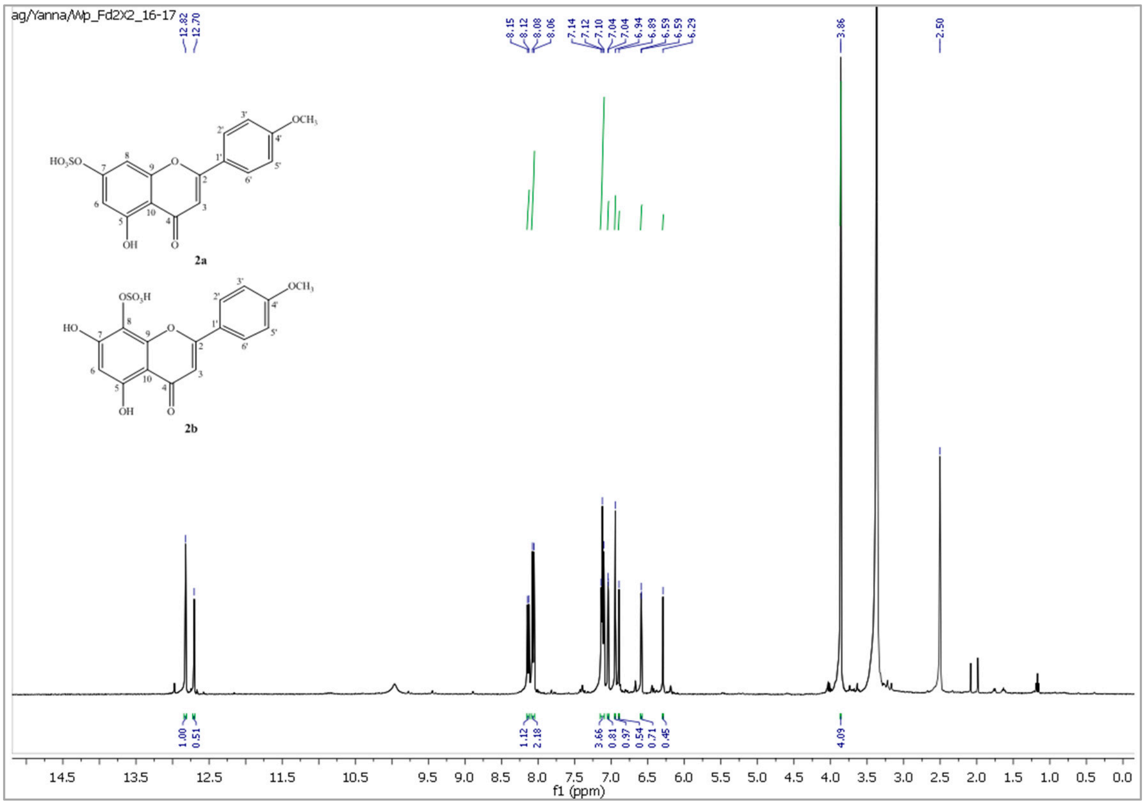


**Figure S8.** ¹H-NMR spectrum (400 MHz, DMSO) of **2a** + **2b**.

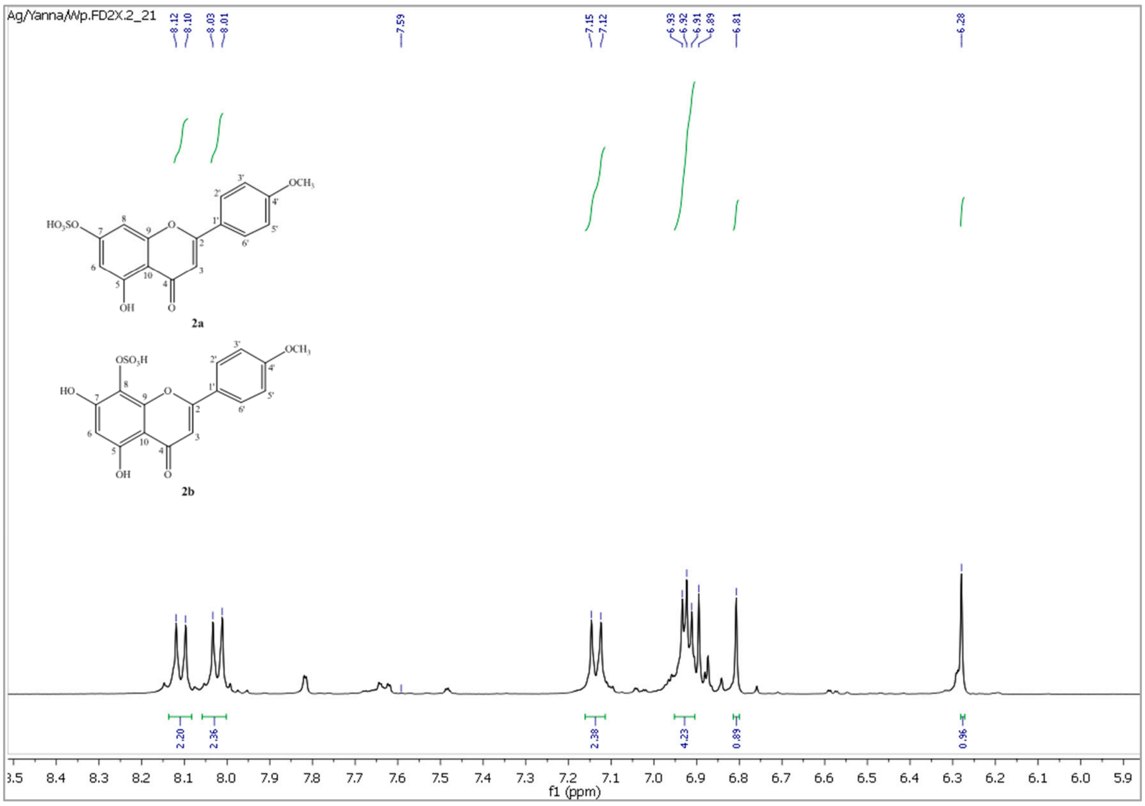


**Figure S9.** Expansion of ¹H-NMR spectrum (400 MHz, DMSO) of **2a** + **2b**.

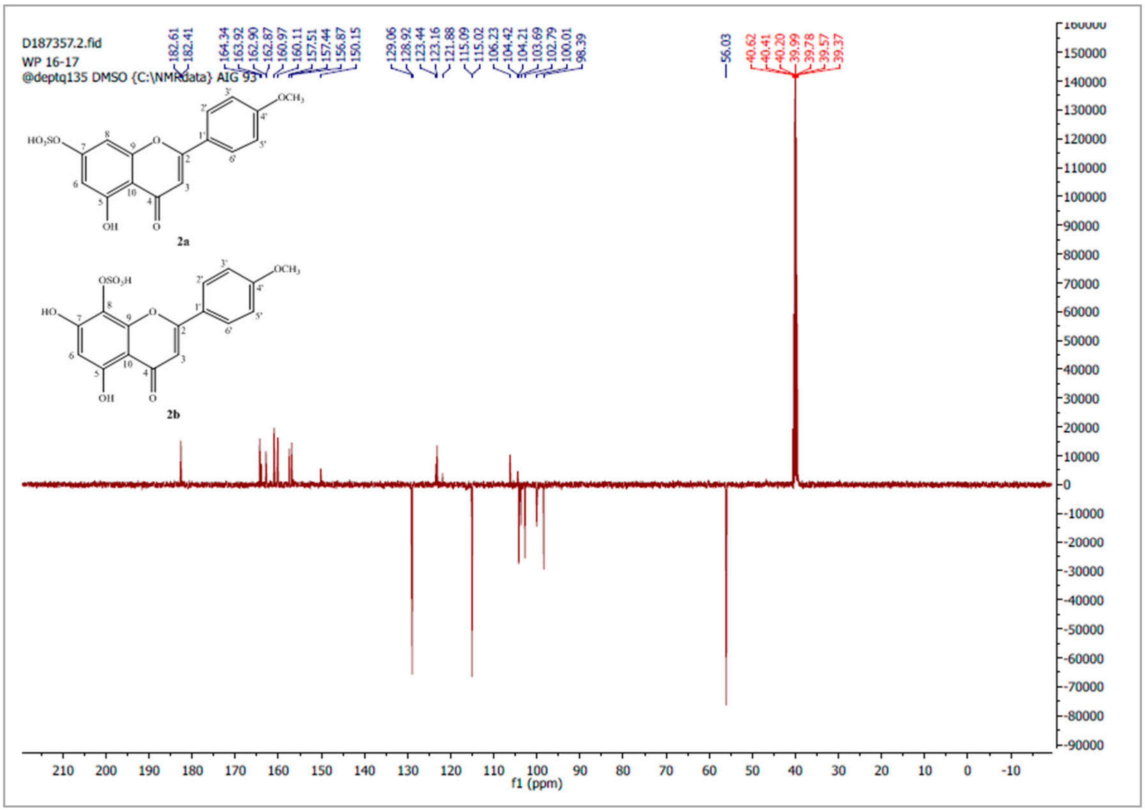


**Figure S10.** $^{13}C$ DEPTQ-135 NMR spectrum (100 MHz, DMSO) of **2a** + **2b**.

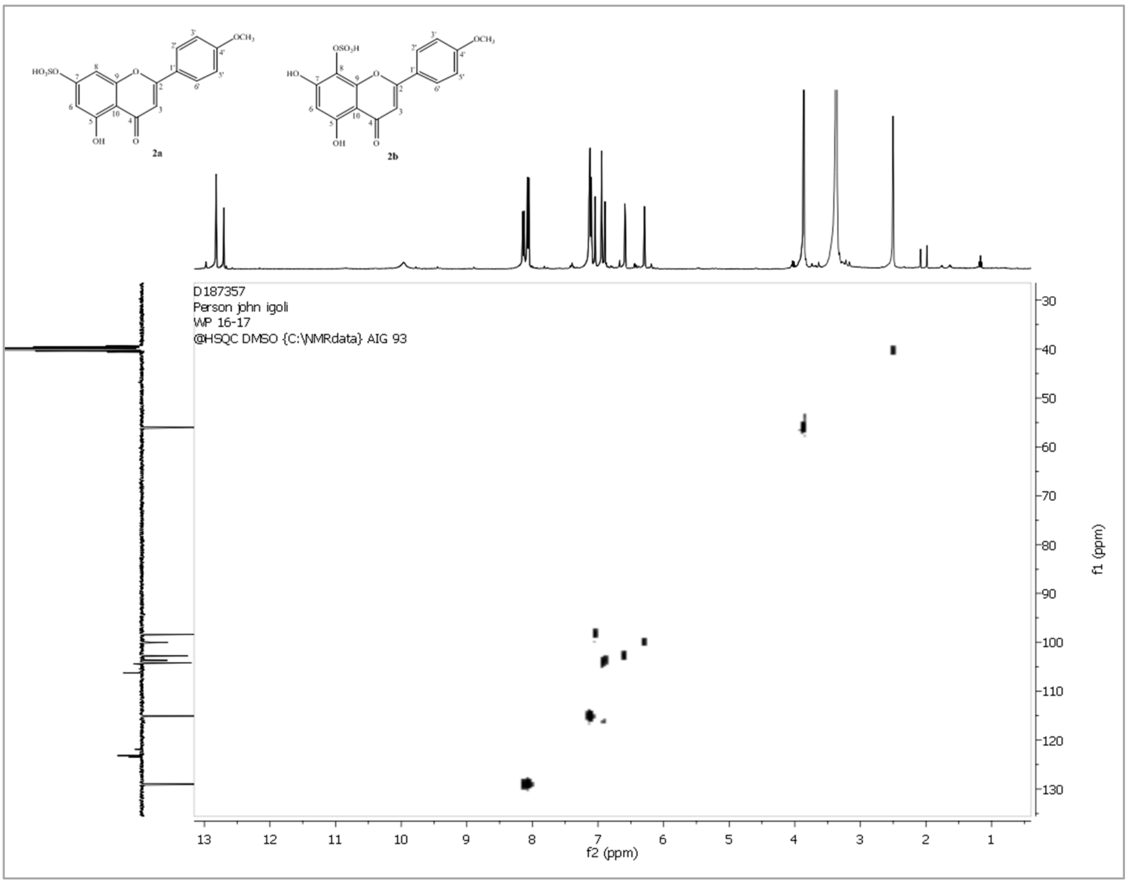


**Figure S11.** HSQC spectrum ($^{1}H$-NMR: 400 MHz, $^{13}C$-NMR: 100 MHz, DMSO) of **2a** + **2b**.

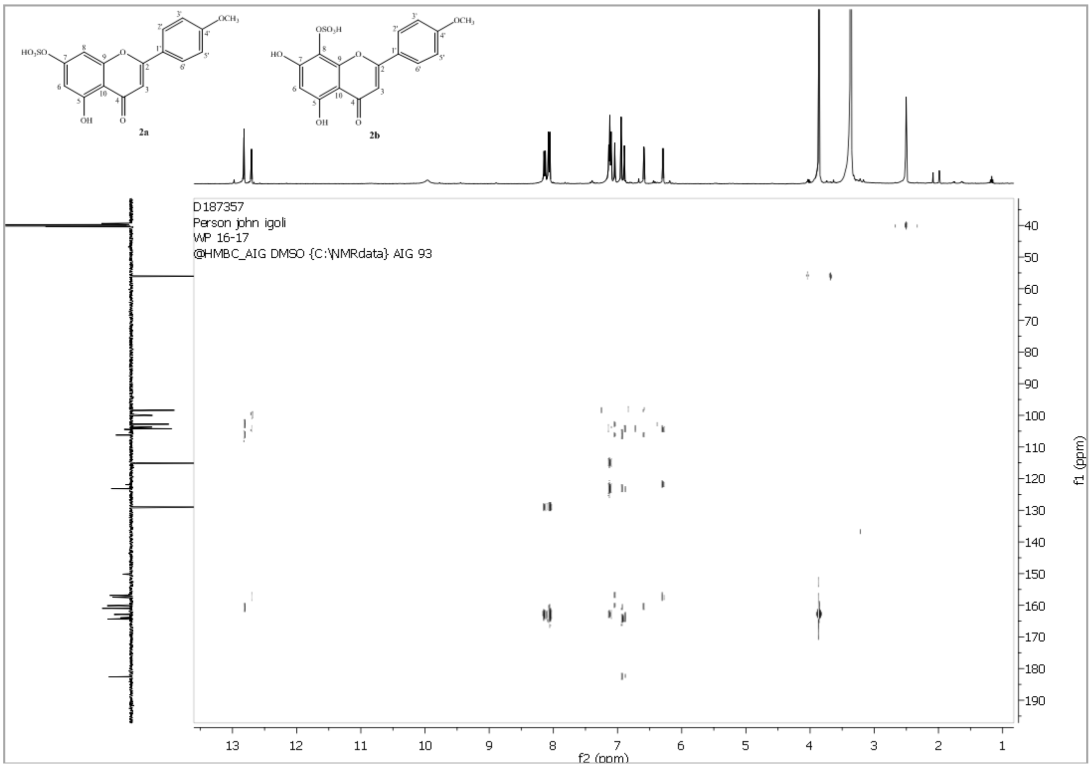


**Figure S12.** HMBC spectrum ($^{1}$H-NMR: 400 MHz, $^{13}$C-NMR: 100 MHz, DMSO) of **2a** + **2b**.

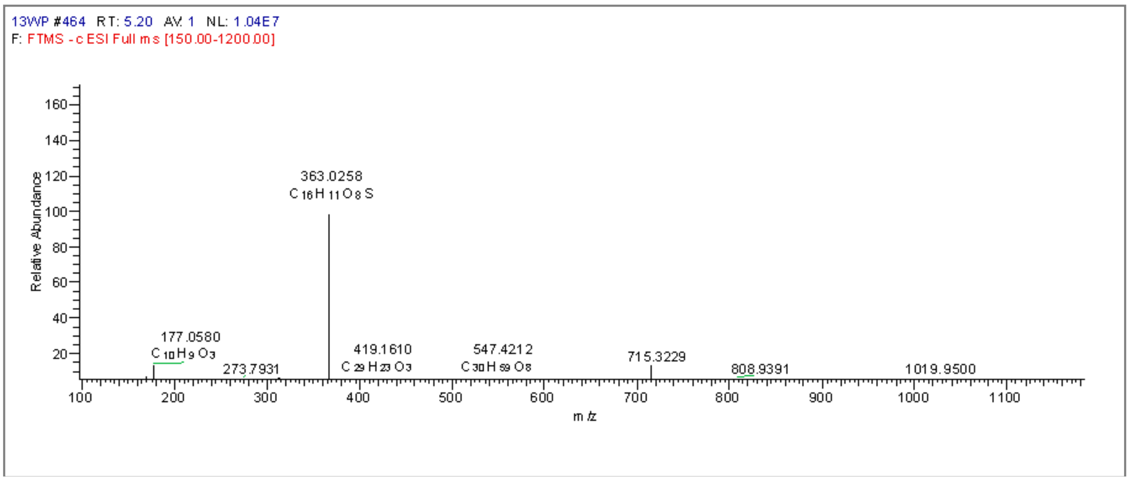


**Figure S13.** HRMS spectrum of compound **2a**.

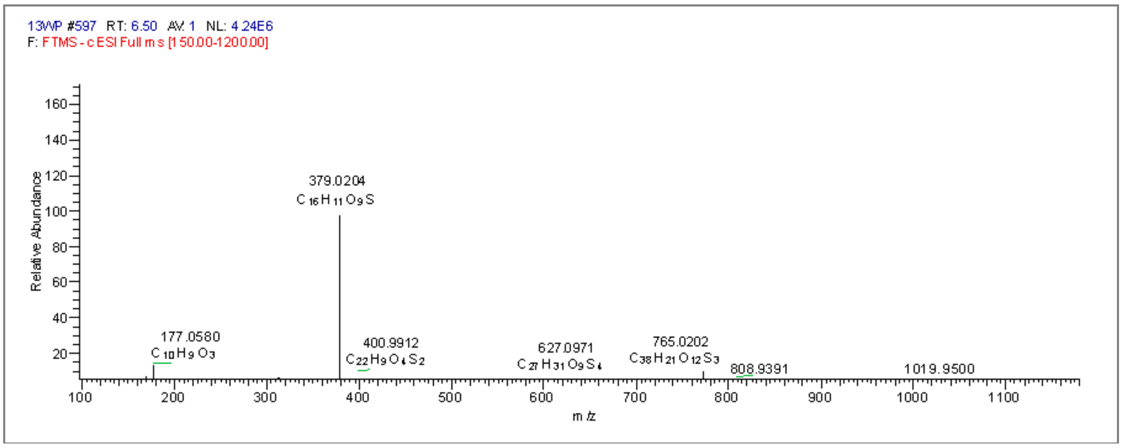


**Figure S14.** HRMS spectrum of compound **2b**.

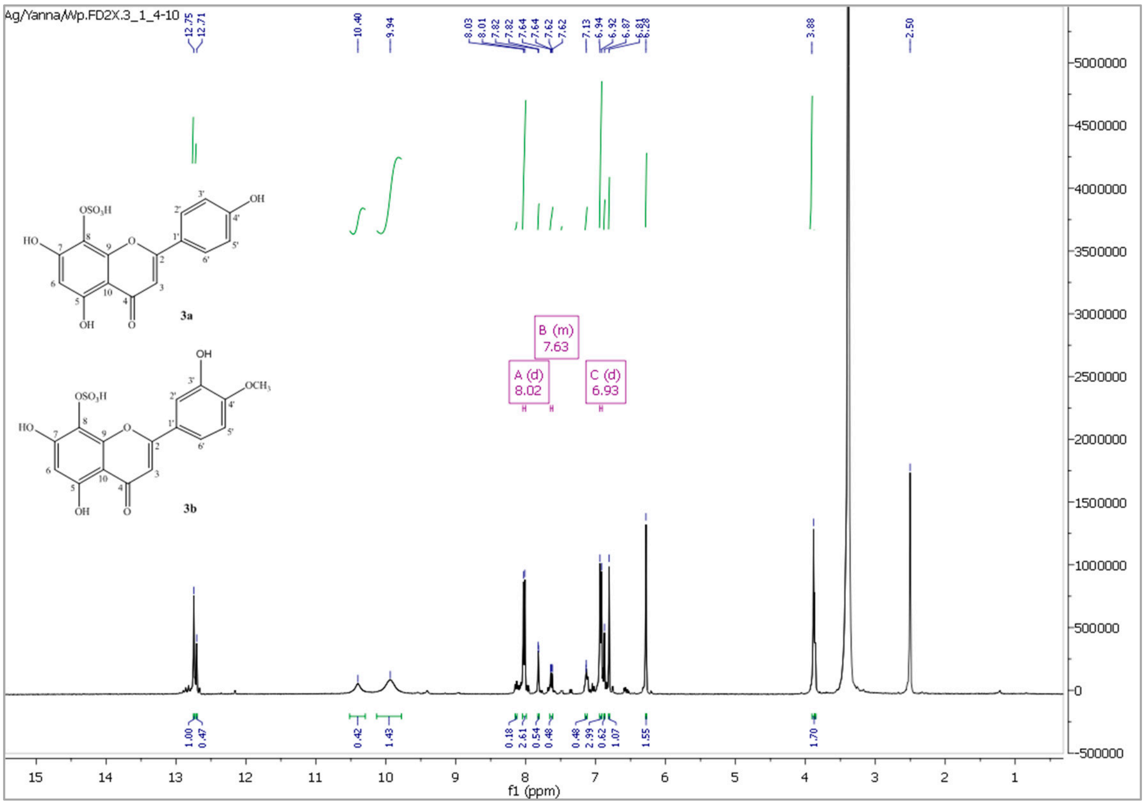


**Figure S15.** $^{1}$H-NMR spectrum (400 MHz, DMSO) spectrum of **3a** + **3b**.

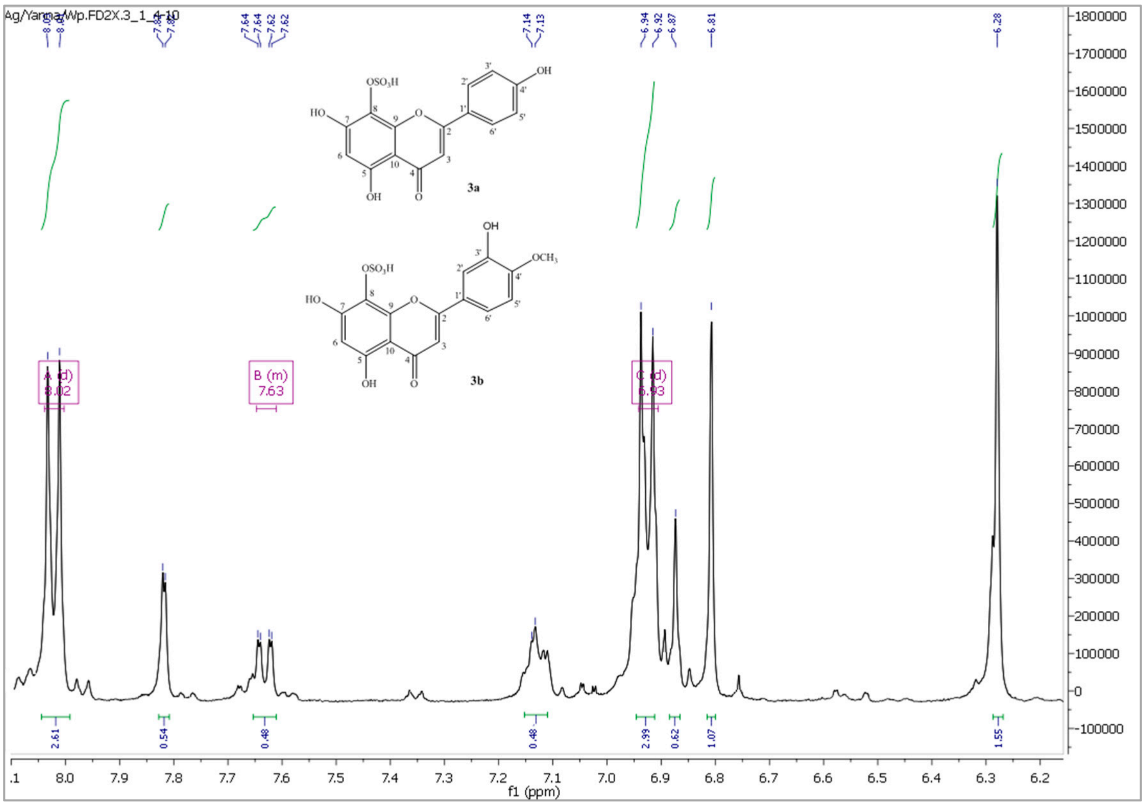


**Figure S16.** Expansion of $^{1}$H-NMR spectrum (400 MHz, DMSO) spectrum of **3a** + **3b**.

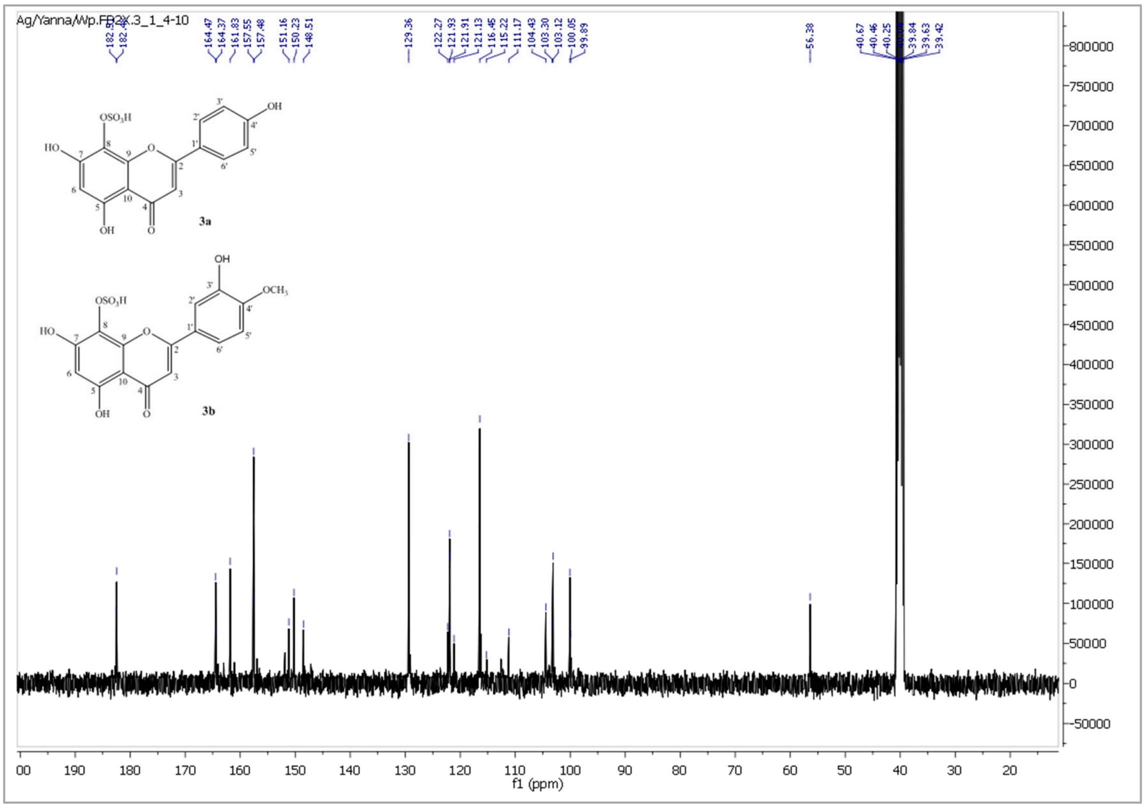


**Figure S17.** $^{13}$C-NMR spectrum (100 MHz, DMSO) spectrum of **3a** + **3b**.

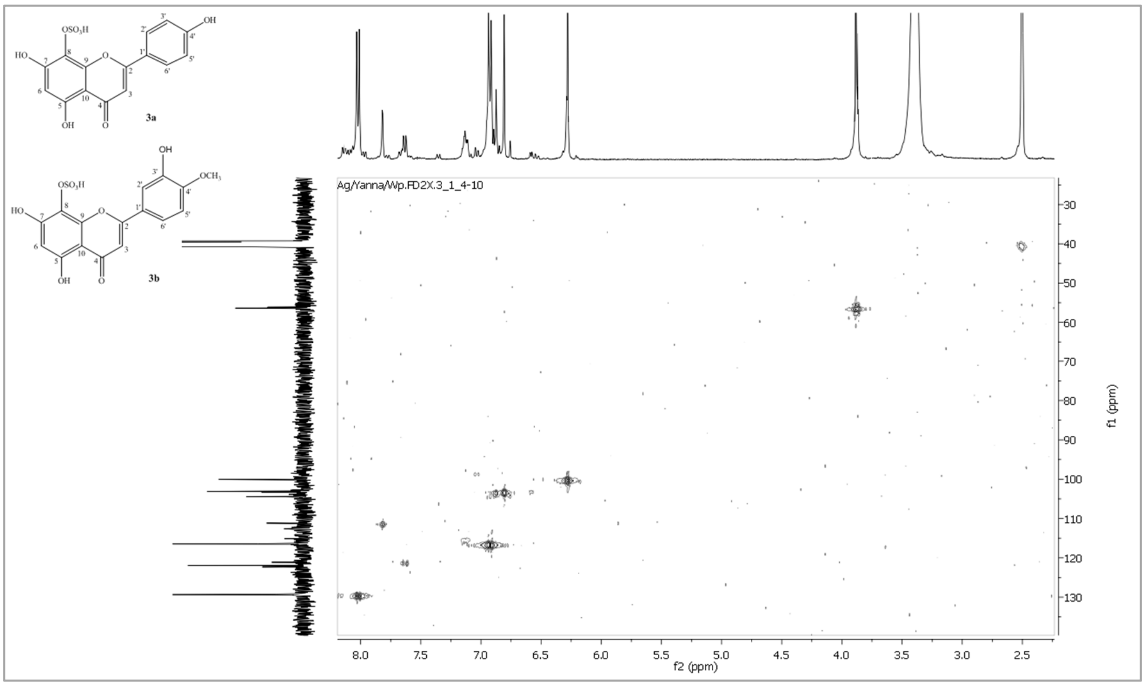


**Figure S18.** HSQC spectrum ($^{1}$H-NMR: 400 MHz, $^{13}$C-NMR: 100 MHz, DMSO) of **3a** + **3b**.

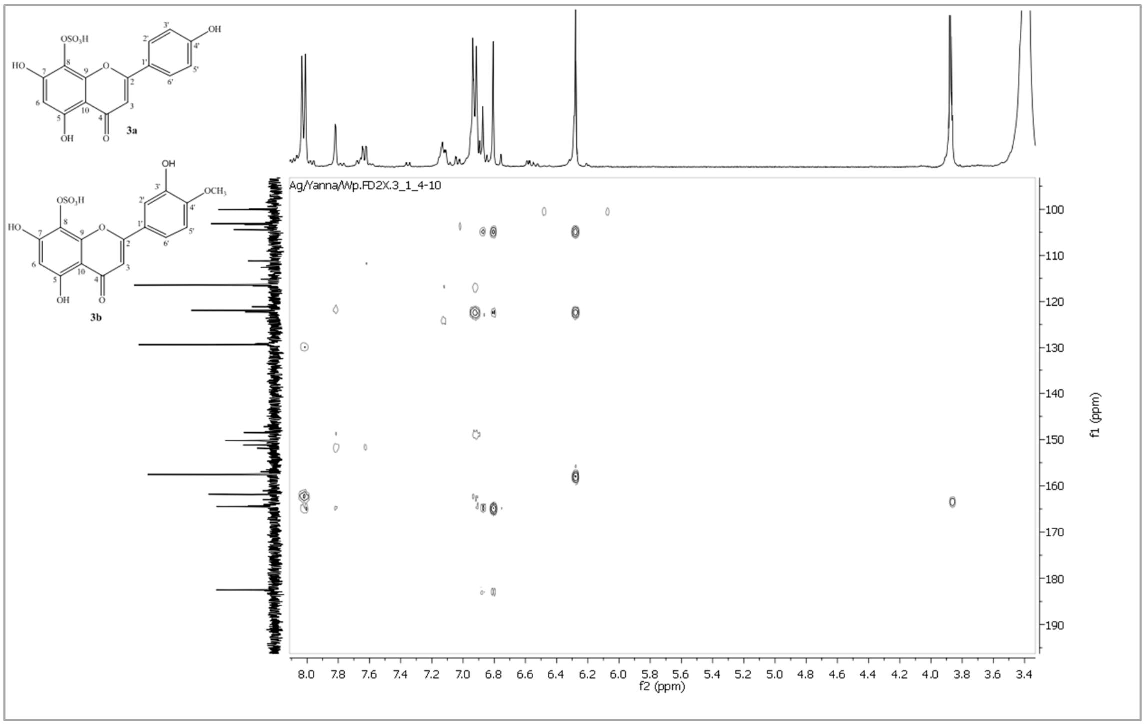


**Figure S19.** HMBC spectrum ($^{1}$H-NMR: 400 MHz, $^{13}$C-NMR: 100 MHz, DMSO) of **3a** + **3b**.

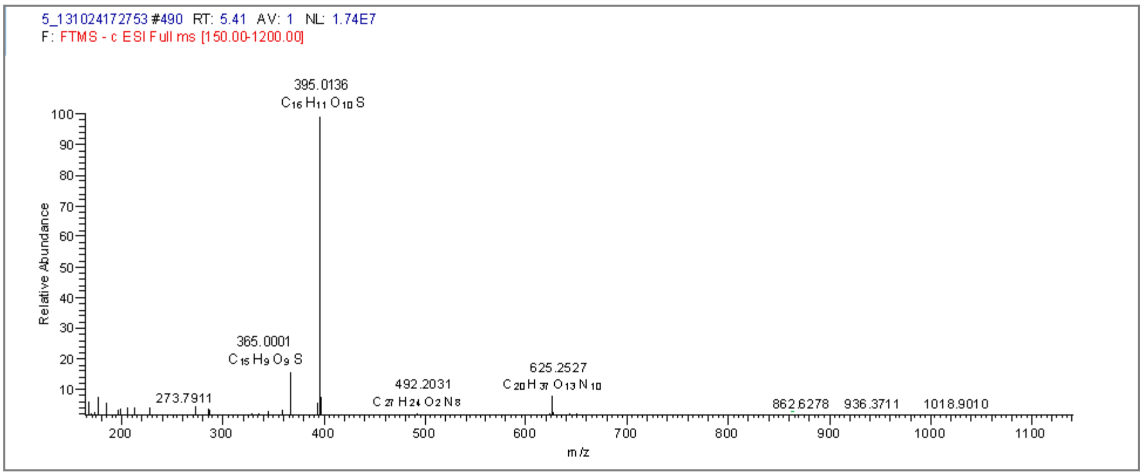


**Figure S20.** HRMS spectrum of compound **3b**.

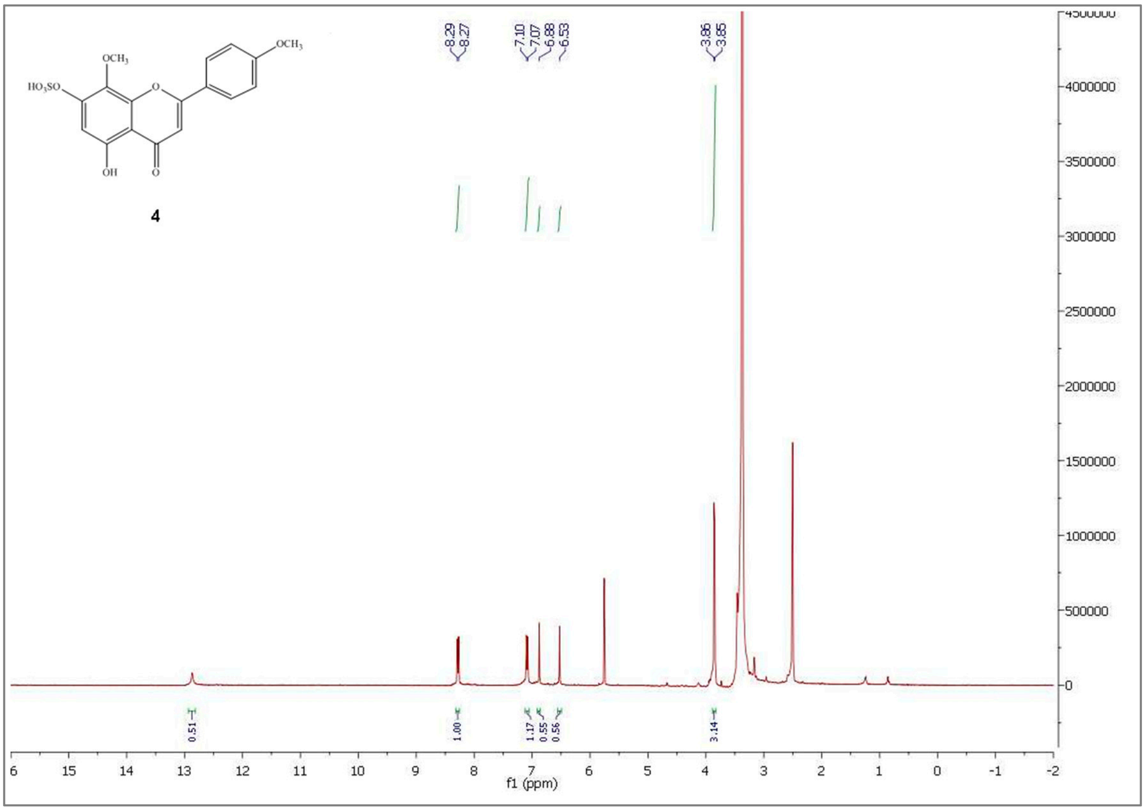

**Figure S21.** $^1$H-NMR spectrum (400 MHz, DMSO) spectrum of **4**.

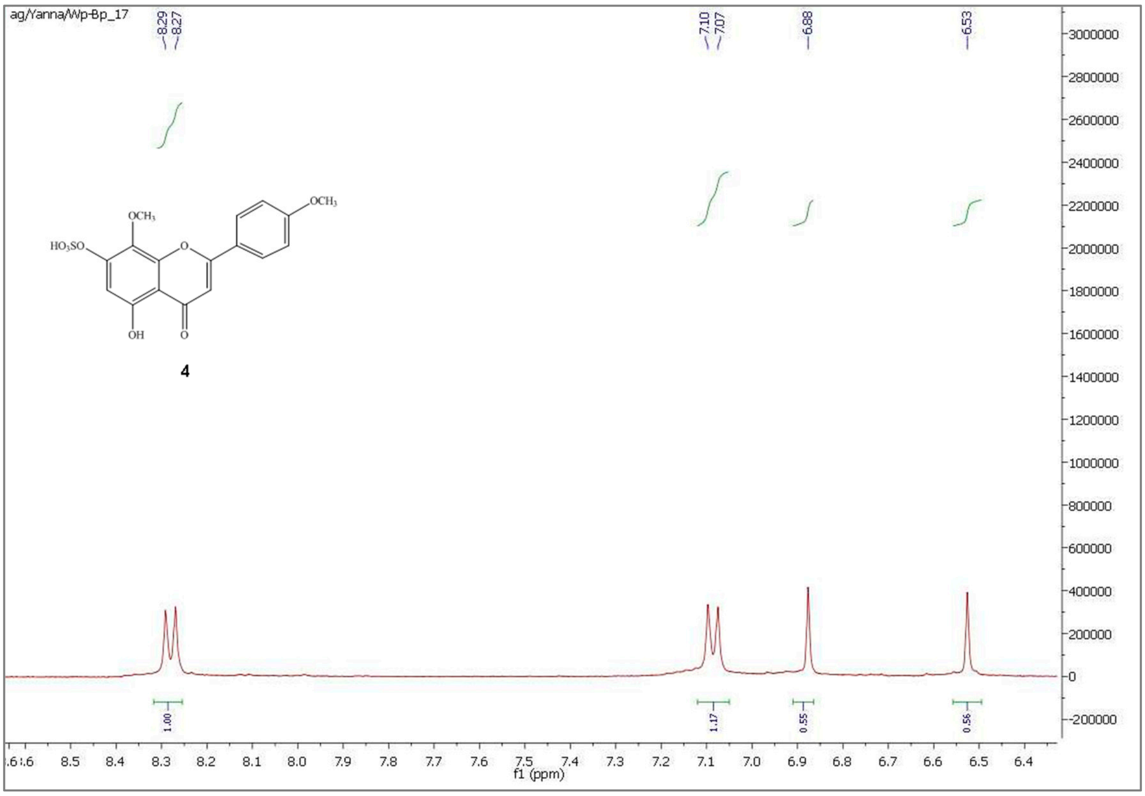

**Figure S22.** Expansion of $^1$H-NMR spectrum (400 MHz, DMSO) spectrum of **4**.

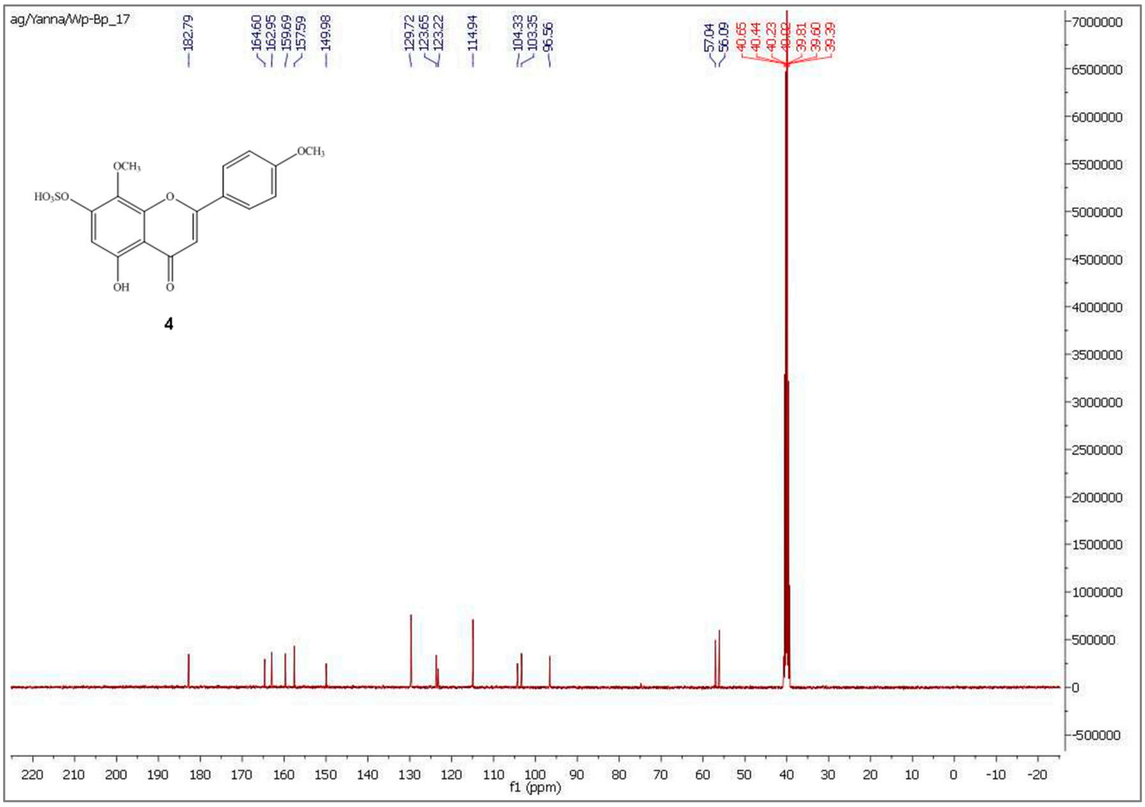


**Figure S23.** $^{13}$C-NMR spectrum (100 MHz, DMSO) spectrum of **4**.

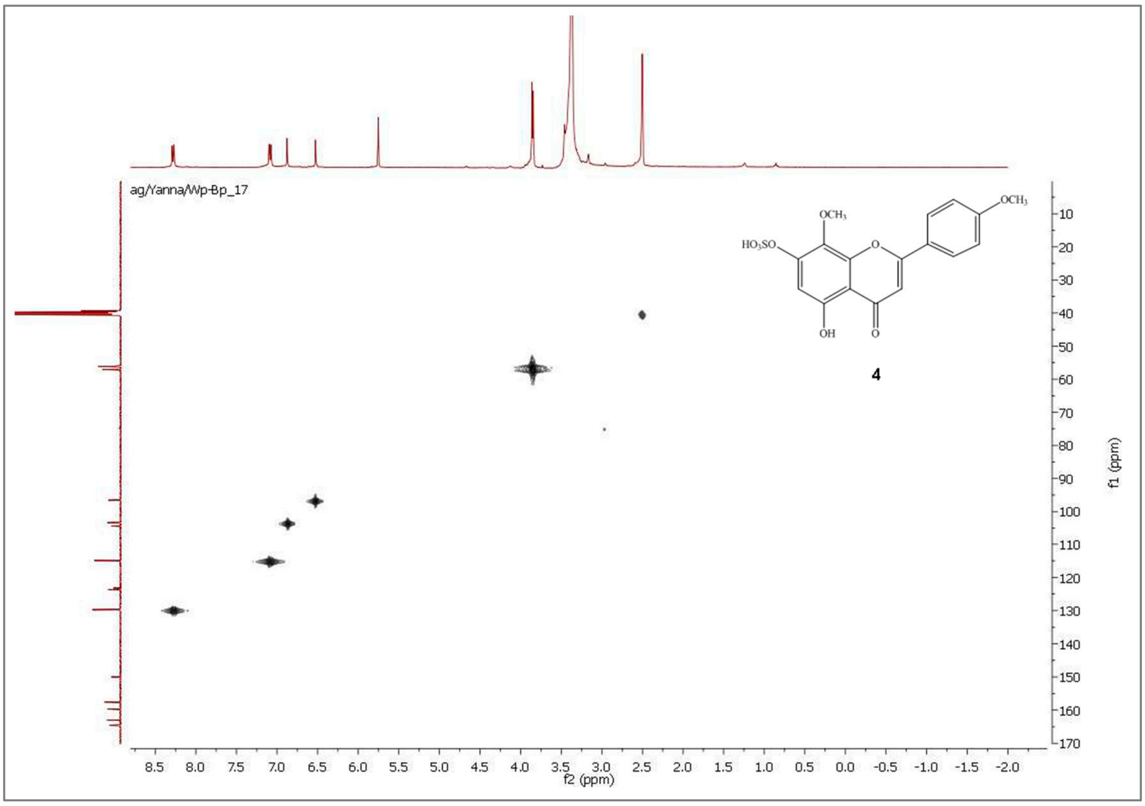


**Figure S24.** HMQC spectrum ($^{1}$H-NMR: 400 MHz, $^{13}$C-NMR: 100 MHz, DMSO) of **4**.

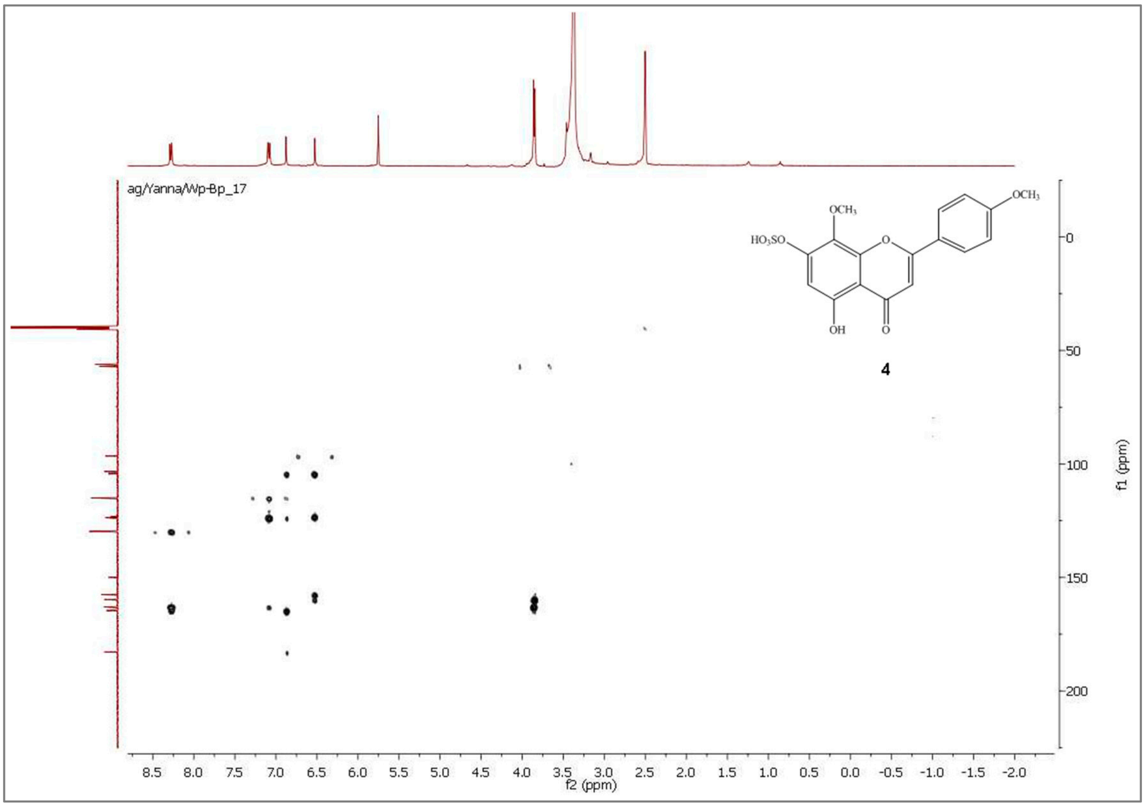


**Figure S25.** HMBC spectrum ($^{1}$H-NMR: 400 MHz, $^{13}$C-NMR: 100 MHz, DMSO) of **4**.

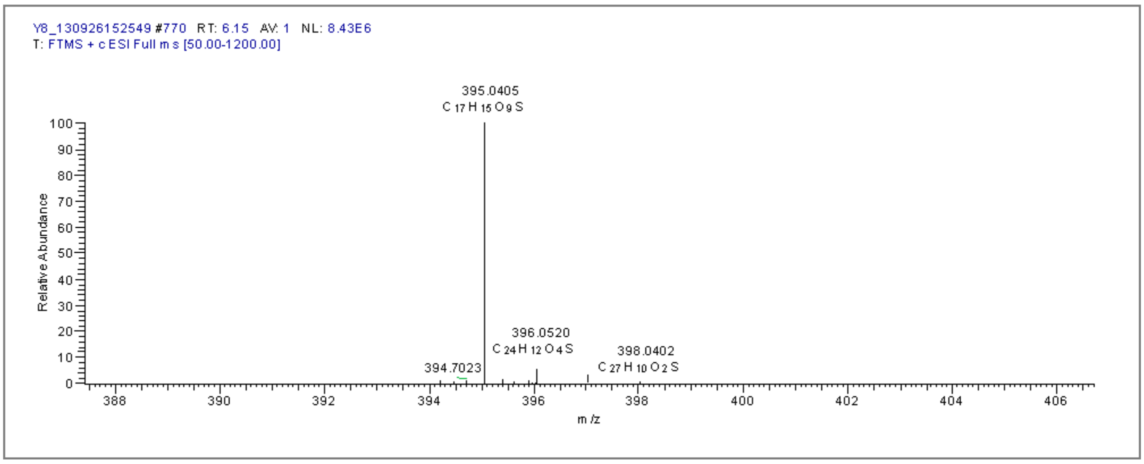


**Figure S26.** HRMS spectrum of compound **4**.
